# Impact of a novel protein meal on the gastrointestinal microbiota and the host transcriptome of larval zebrafish *Danio rerio*

**DOI:** 10.3389/fphys.2015.00133

**Published:** 2015-04-30

**Authors:** Eugene Rurangwa, Detmer Sipkema, Jeroen Kals, Menno ter Veld, Maria Forlenza, Gianina M. Bacanu, Hauke Smidt, Arjan P. Palstra

**Affiliations:** ^1^Institute for Marine Resources and Ecosystem Studies, Wageningen University and Research CentreYerseke, Netherlands; ^2^Laboratory of Microbiology, Wageningen UniversityWageningen, Netherlands; ^3^Aquaculture and Fisheries Group, Wageningen UniversityWageningen, Netherlands; ^4^Cell Biology and Immunology Group, Wageningen UniversityWageningen, Netherlands

**Keywords:** zebrafish nutrition, 16S rRNA-based microbial composition, pyrosequencing, mRNA sequencing, gastrointestinal tract transcriptome, iron metabolism, aquaculture

## Abstract

Larval zebrafish was subjected to a methodological exploration of the gastrointestinal microbiota and transcriptome. Assessed was the impact of two dietary inclusion levels of a novel protein meal (NPM) of animal origin (ragworm *Nereis virens*) on the gastrointestinal tract (GIT). Microbial development was assessed over the first 21 days post egg fertilization (dpf) through 16S rRNA gene-based microbial composition profiling by pyrosequencing. Differentially expressed genes in the GIT were demonstrated at 21 dpf by whole transcriptome sequencing (mRNAseq). Larval zebrafish showed rapid temporal changes in microbial colonization but domination occurred by one to three bacterial species generally belonging to *Proteobacteria* and *Firmicutes*. The high iron content of NPM may have led to an increased relative abundance of bacteria that were related to potential pathogens and bacteria with an increased iron metabolism. Functional classification of the 328 differentially expressed genes indicated that the GIT of larvae fed at higher NPM level was more active in *transmembrane ion transport* and *protein synthesis*. mRNAseq analysis did not reveal a major activation of genes involved in the immune response or indicating differences in iron uptake and homeostasis in zebrafish fed at the high inclusion level of NPM.

## Introduction

The diet has profound effects on the microbial composition and on the nutrient uptake by the enterocytes in the GIT. Moreover, the diet has effects on the interactions between host and microbes, aspects of which can be very specific (Rawls et al., 2004) but which is also surprisingly conserved across all vertebrates (Rawls et al., 2006). During long-lasting interactions, coevolution between hosts and microbes has resulted in a microbial ecosystem that is monitored and controlled by the host while the microbiota influence their host to maintain a stable niche for its continued presence (Neish, 2009).

Commensal microbial communities play an important role in the host's GIT development, nutrition and protection against pathogens (Verschuere et al., 2000; Bates et al., 2006; Nayak, 2010; Ringø et al., 2010). In the absence of microbes (germ-free fish), specific aspects of GIT differentiation and functions are arrested or altered (Bates et al., 2006). GIT microbiota are involved in the host's feed digestion and physiological processes by producing vitamins, digestive enzymes, amino acids, essential growth factors and metabolites (Nayak, 2010). They affect a wide range of biological processes including nutrient processing and absorption, regulation of intestinal glycan expression, development of the mucosal immune system and fortification of the innate immune defenses, angiogenesis, and epithelial renewal (reviewed by Rawls et al., 2004; Kanther and Rawls, 2010).

Zebrafish offers interesting features as model organism to study the nutritional impact of alternative protein sources on the GIT functions, development of the microbial community and host—microbe interactions by combining several molecular based approaches. Key features of the zebrafish model include a characterized genome, a wide variety of molecular and bioinformatic tools and a well-characterized rapid embryonic development (Westerfield, 1993; Kimmel et al., 1995; Ulloa et al., 2011). With these advantages related to the use of zebrafish as an experimental fish model, nutritional research in aquaculture can be conducted at reduced cost, time and space needed in research facilities (Gomez-Requeni et al., 2010; Ribas and Piferrer, 2013). Zebrafish thereby offers an opportunity to gain mechanistic insights but, as any model, does not replace the commercial species of interest that has its own GIT characteristics.

When kept at 28°, zebrafish larvae hatch from their chorions within 3 days post-fertilization (dpf), and the mouth opens around 74 h post-fertilization (hpf). The GIT is colonized by microbiota from the environment after hatching within 12–24 h, concurrent with digestive tract differentiation (Hansen and Olafsen, 1999; Bates et al., 2006; Rawls et al., 2007; Nayak, 2010). By 4 dpf, within a day after mouth opening, the digestive tract is colonized by a small number of bacteria and their number increases after swallowing has started (Bates et al., 2006). At 5 dpf, the GIT is fully functional from a nutritional point of view when lipid and protein macromolecule uptake is apparent (Farber et al., 2001; Wallace et al., 2005), a regular pattern of spontaneous movement is visible and exogenous feeding commences (Holmberg et al., 2004). The yolk is largely absorbed and GIT morphogenesis has proceeded to a stage that supports feeding and digestion (Farber et al., 2001; Rawls et al., 2004).

This study aimed to determine the impact of a novel protein meal (NPM) on the gastrointestinal microbiota and the host transcriptome of larval zebrafish. The NPM that was tested is of animal origin (ragworm *Nereis virens*) and has been demonstrated to be beneficial for hematocrit levels and general physiological performance as suggested by improved growth in common sole Solea solea when compared to fish fed with commercial pelleted feeds (Kals, 2014). Therewith it potentially has considerable importance for aquaculture nutrition. The present study was undertaken to assess the impact of two dietary inclusion levels of the NPM on the GIT of the developing zebrafish, specifically on (1) microbial development over the first 21 dpf, and (2) molecular differentiation in physiological processes in the host by differentially expressed genes at 21 dpf as determined by mRNAseq. These investigations are performed with an unbiased approach.

Pathogens residing in the GIT are known to be stimulated in their pathogenic potential by increased iron availability (Kortman et al., 2012). As feed ingredients of animal origin are expected to be rich iron sources, a bias was introduced to particularly assess (1) changes in the abundance of potential pathogens and consequences for the expression of GIT genes involved in immune response, and (2) changes in the abundance of bacteria with increased iron metabolism and consequences for the expression of GIT genes involved in iron uptake and homeostasis. It is hypothesized that increasing the dietary inclusion level of the NPM will lead to (1) a higher abundance of potential pathogens and bacteria with an increased iron metabolism, and (2) differential expression of genes indicating an activation of the immune response, lower iron uptake and increased attention for maintaining homeostasis.

## Methods

### Zebrafish husbandry

**Figure 1:**
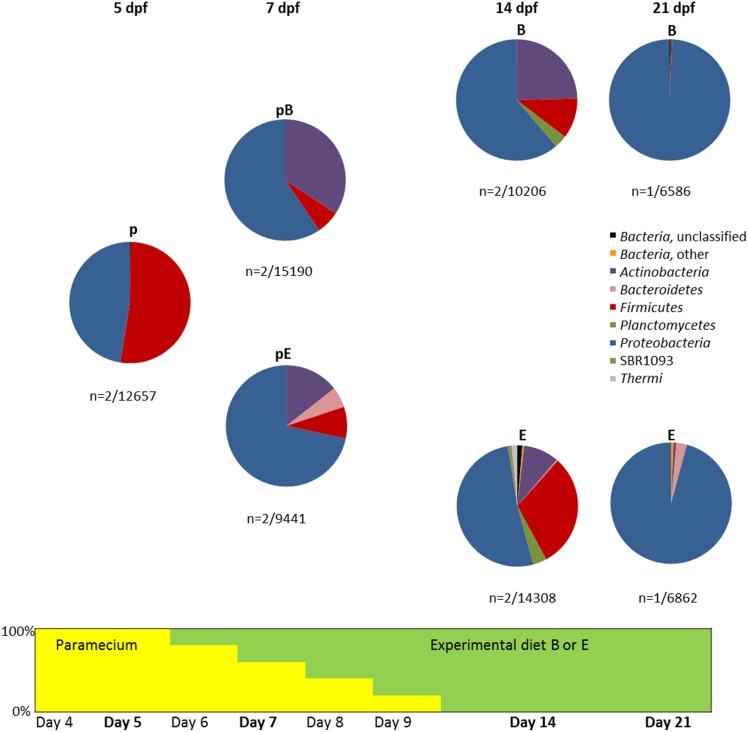
**Taxonomic distribution of bacterial 16S rRNA gene reads retrieved from zebrafish raised with different diets during the first 21 dpf**. Only phyla that represent more than 1% of the reads in at least one of the samples are shown and chloroplasts-affiliated reads were removed prior to analysis. The pie diagrams shown are averages of replicate samples with the number of samples and total number of reads below each pie chart. Not all replicate samples that were initially taken resulted in successful DNA extraction due to the small sample size. The step-wise decrease of *Paramecium* in the diet is indicated below the pie charts, with the percentage of *Paramecium* in yellow and the percentage of Diet B/E in green.

Zebrafish (*Danio rerio* Hamilton 1822) embryos were obtained from breeders of the Zod2F7 strain. The ancestral diet consisted of live nauplii of *Artemia* (brine shrimp) and commercial flake diet for ornamental fish (Tetra). At 5 dpf, 1800 larvae from a single batch were randomly distributed over 6 experimental aquaria (each 6 L with 50 individuals per liter) installed in a thermo-regulated water bath and with individual inflow water connected to a flow-through system. Larvae were reared at a photoperiod of 14/10 h light/dark and under optimal water quality conditions (Temperature 25.9 ± 0.3°C; pH 8.1 ± 0.1; dissolved oxygen 7.6 ± 0.3 mg/L; ammonium, nitrogen and nitrate null; nitrite concentration 0.00–0.05 mg/L). Larvae were fed *Paramecium* (diet p) at 4 and 5 dpf, then gradually weaned to experimental diets between 6 and 9 dpf (diets pB and pE; Figure 1) and, from 10 dpf onwards, fed with experimental diets (diets B and E) until 21 dpf. During the transitional feeding period from live to inert dry feed, larvae were fed a daily ration of paramecium decreasing with 20% per day. Before live prey had disappeared in the tanks, feeding was completed with an increasing amount of experimental dry feeds.

### Experimental diets

The experimental diets were isonitrogenous, isoenergetic, equal in amino acids composition, calcium and phosphates, but differed in concentration of the NPM ragworm (*Nereis virens*) meal (Seabait Ltd, Woodhorn Village, UK). Diet B (10% NPM) and E (75% NPM) were fed by hand till satiation 4–7 times per day. The dry micro-particulate lyophilised diets were prepared through cold extrusion (Research Diet Services, Wijk bij Duurstede, the Netherlands). 200 μm micro-particulates were fed between 6 and 10 dpf and 200–300 μm between 11 and 20 dpf for both experimental diets. The crude composition (Table 1) was analyzed at Nutrilab bv (Giessen, the Netherlands) and the iron content was analyzed at the Chemical Biological Soil Laboratory (Wageningen, the Netherlands) using Inductively Coupled Plasma Atomic Emission Spectroscopy.

**Table 1:** **Experimental diets**.

**Amount of novel protein meal (%)**	**Dietary treatment**			
	**10**^1^		**75**^1^	
**Code**	**B**		**E**	
**INGREDIENTS IN %**				
Novel protein meal^a^	10.00		75.00	
Pea protein^b^	21.88		7.94	
Casein^c^	19.42		3.66	
Soy Protein Concentrate^d^	15.20		0.00	
Fish Oil^e^	10.24		0.00	
Diamol^f^	8.33		0.00	
Sugar^g^	1.32		0.00	
Lime^h^	0.20		0.00	
Wheat gluten^i^	5.00		5.00	
Binder 1^j^	2.00		2.00	
Binder 2^k^	2.00		2.00	
Salt^l^	2.00		2.00	
Binder 3^m^	1.00		1.00	
Mineral and vitamin premix^†^	1.36		1.36	
Betaine^n^	0.05		0.05	
**Calculated (composition)**	**B**		**E**	
DM (g.kg^−1^)	921.7		941.9	
ASH (g.kg^−1^)	144.5		157.4	
CP (g.kg^−1^)	533.2		533.1	
EE (g.kg^−1^)	134.8		134.8	
Ca (g.kg^−1^)	2.4		2.3	
P (g.kg^−1^)	5.6		6.1	
GE	21.4		21.2	
CP/GE	25.9		26.2	
Iron (mg.kg^−1^)	312		1486	
**CALCULATED AMINO ACIDS (g.kg^−1^)**				
Lysine^*^	37.1		34.1	
Methionine^*^	10.3		10.3	
Cysteine^**^	5.6		5.7	
Threonine^*^	21.2		19.7	
Tryptophan^*^	6.3		6.1	
Isoleucine^*^	26.0		21.8	
Arginine^*^	32.9		32.8	
Phenylalanine^*^	27.6		22.0	
Histidine^*^	14.5		13.4	
Leucine^*^	45.2		37.0	
Tyrosine^**^	21.6		19.1	
Valine^*^	29.7		25.5	
Alanine	22.2		34.1	
Asparagine	50.8		44.2	
Glutamate	106.0		78.7	
Glycine	18.9		26.3	
Proline	40.1		38.3	
**CALCULATED AMINO ACIDS (g.kg−1)**	**B**		**E**	
Serine	27.0		21.0	
**ANALYZED (COMPOSITION)**				
**Size of feed**	**200 μm**	**200–300 μm**	**200 μm**	**200–300 μm**
DM (g.kg^−1^)	960	956	961	948
ASH (g.kg^−1^.dm)	164	159	185	184
CP (g.kg^−1^.dm)	573	586	556	572
EE (g.kg^−1^.dm)	122	120	103	156
GE	20.9	21.0	20.0	21.3
CP/GE	27.4	27.9	27.8	26.9

1 *Percentage of novel protein meal: Recipes are isoenergetic. Composition of diet B and E are equal in macronutrients, amino acids, calcium and phosphates. The novel protein meal contains 17 percent of fat (ether extract) of which the composition is comparable to that of fish oil as the novel protein meal is made of a marine invertebrate. Calculated omega 3 content of diet B and E were 21.3 and 19.5 g.kg^–1^.dm^−1^ respectively*.

† *vitamins (mg or IU kg^−1^ diet) include: vitamin A (retinyl acetate), 2.4 mg, 8000 IU; vitamin D3 (cholecalciferol), 0.04 mg, 1700 IU; vitamin K3 (menadione sodium bisulfite), 10 mg; vitamin B1 (thiamine), 8 mg; vitamin B2 (riboflavin), 20 mg; vitamin B6, vitamin B12 (cyanocobalamin) 0.02 mg (pyridoxine hydrochloride), 10 mg; folic acid, 6 mg; biotin, 0.7 mg; inositol, 300 mg; niacin, 70 mg; pantothenic acid, 30 mg, choline, 1500 mg; vitamin C, 500 mg; vitamin E, 300 mg; Minerals (g or mg kg^−1^ diet): Mn (manganese oxide), 20 mg; I (potassium iodide), 1.5 mg; Cu (copper sulfate), 5 mg; Co (cobalt sulfate), 0.1 mg; Mg (magnesium sulfate), 500 mg; Zn (zinc oxide), 30 mg; Se (sodium selenite), 0.3 mg; Fe (Iron Sulfate), 60 mg; Calcium carbonate, 2150 mg; Dicalcium phosphate, 5000 mg; Potasium Chloride, 1000 mg; Antixoidant BHT (E300-321), 100 mg; Anti-fungal Calcium propionate, 1000 mg*.

a *Ingredient is not specified because of confidentiality reasons of ongoing research*.

b *Roquette Freres, Lestrem, France*.

c *Acid casein 30/60 mesh, Lactalis, Bourgbarré, France*.

d *Soycomil R ADM Eurpoort BV, the Netherlands*.

e *Coppens International, the Netherlands*.

f *Damolin A/S, Hamburg, Germany*.

g *Melis Suikerunie, Dinteloord, the Netherlands*.

h *Inducal 250, Sibelco/Ankerpoort, Maastricht, the Netherlands*.

i *Gluvital 21,000, Cargill, Bergen op Zoom, the Netherlands*.

j *Binder1*.

k *Binder2*.

l *Animalfeed salt, Kloek zout, the Netherlands*.

m *Binder3: Ingredients are not specified because of confidentiality reasons of ongoing research*.

n *Betafin, Danisco Animal Nutrition Marlborough UK*.

† *vitamins (mg or IU kg^−1^ diet) include: vitamin A (retinyl acetate), 2.4 mg, 8000 IU; vitamin D3 (cholecalciferol), 0.04 mg, 1700 IU; vitamin K3 (menadione sodium bisulfite), 10 mg; vitamin B1 (thiamine), 8 mg; vitamin B2 (riboflavin), 20 mg; vitamin B6*.

* *essential*.

** *conditionally essential*.

### Larval sample collection and storage

Larvae were sacrificed by an overdose of the anesthetic 1.0% tricaine methane sulfonate buffered with 1.5% NaHCO3. Triplicate pools of 10 larvae per diet were collected in sterile condition at 5, 7, 14, and 21 dpf. External surfaces of larvae were disinfected by rinsing with 70% ethanol for 2 min and then several times with sterile filtered (0.2 micron) Milli-Q water. In addition, at 21 dpf, extra triplicate pools of 10 larvae per diet were collected for GIT sampling. Larvae were anesthetized, disinfected, and dissected on ice in sterile conditions using flamed instruments between two different samples. Whole larvae were kept frozen in sterile Eppendorff tubes at −20°C directly upon sampling and were then stored at −80°C, while extra GIT samples from day 21 were stored in RNAlater (Applied Biosystems, Nieuwerkerk a/d lJssel, the Netherlands) at −20°C.

### Microbiological analyses: DNA isolation

Microbial DNA was isolated using the protocol described by Roeselers et al. (2011) with some modifications: Ten larvae were combined in 2.0 ml screw-cap tubes containing 0.1 mm Zirconia/silica beads and 2.5 mm Glass beads (Biospec Products). 800 μl 120 mM Na-phosphate buffer (pH 8.0) and 400 μl of lysis solution containing 10% sodium dodecyl sulfate, 0.5 M Tris-HCl (pH 8.0) and 0.1 M NaCl was added homogenisation in a Mini-beadbeater (Biospec Products) for 6 min at 5500 rpm. The supernatants were transferred to new tubes and lysozyme was added to a final concentration of 10 mg/ml followed by incubation at 42°C for 30 min. Ammonium acetate (7.5 M) was added to the supernatant (2:5 v/v) and samples were incubated at −20°C for 5 min. Samples were centrifuged for 5 min at 12,000 g and the supernatants were transferred to new tubes. DNA was precipitated at room-temperature with isopropyl alcohol (500 μl) and pelleted by centrifugation at 12,000 rpm for 30 min at 4°C. Pellets were washed with −20°C 70% ethanol and air-dried for 45 min before resuspension in 50 μl nuclease free water.

### Microbiological analyses: RT-PCR

RT-PCR targeting 16S rRNA was performed with the primers 27F and 1492R (Lane, 1991) for total RNA extracted from two pools of GIT for both diet B and diet E (for RNA extraction procedure see section “*mRNAseq: Total RNA isolation*”). The RT reaction (20 μl) contained 50 mM Tris-HCl, 75 mM KCl, 3 mM MgCl_2_, 5 μM DTT, 0.5 mM of each dNTP, 2 pmol of primers 27F and 1492R, 200 U of of SuperScript™ Reverse Transcriptase (Invitrogen), 40 U of RNasin® Plus RNase Inhibitor (Promega) and 1 μg of RNA extracted from 10 pooled larvae or GITs for each of the two diets. Reactions were incubated at 55°C for 60 min, followed by 15 min at 70°C to denature the reverse-transcriptase.

### Microbiological analyses: PCR and sequencing

For 16S rRNA gene-based microbial composition profiling, barcoded amplicons from the V1-V2 region of 16S rRNA genes were generated from all DNA and reverse transcribed RNA samples by PCR using the 27F-DegS primer that was appended with the titanium sequencing adaptor A and an 8 nt sample-specific barcode at the 5′ end, and an equimolar mix of two reverse primers (338R I and II), that carried the titanium adaptor B at the 5′ end^58^.

PCRs were performed using a thermocycler GS0001 (Gene Technologies, Braintree, U.K.) in a total volume of 100 μl containing 1 × HF buffer (Finnzymes, Vantaa, Finland), 2 μl 10 mM (each nucleotide) PCR Grade Nucleotide Mix (Roche, Diagnostics GmbH, Mannheim, Germany), 2 units of Phusion® Hot Start II High-Fidelity DNA polymerase, 500 nM of a forward and reverse primer mix (Biolegio BV, Nijmegen, the Netherlands), and 0.2–0.4 ng/μl of template DNA (or cDNA). The amplification program consisted of an initial denaturation at 98°C for 30 s, 35 cycles of: denaturation at 98°C for 10 s, annealing at 56°C for 20 s and elongation at 72°C for 20 s, and a final extension at 72°C for 10 min. PCR products were purified with the High Pure Cleanup Micro Kit (Roche) using 10 μl nuclease-free water for elution, and quantified using a NanoDrop ND-1000 spectrophotometer. Purified PCR products were mixed in equimolar amounts and run on an agarose gel, followed by excision and purification by the DNA gel extraction kit (Millipore, Billerica, MA, USA). Purified amplicon pools were pyrosequenced using a Genome Sequencer FLX in combination with titanium chemistry (GATC-Biotech, Konstanz, Germany). Pyrosequencing data were deposited at the European Bioinformatics Institute in the sequence read archive under study accession number PRJEB4784 and sample accession numbers ERS362581–ERS362592 and ERS362595–ERS362598.

### Microbiological analyses: sequence analysis

Pyrosequencing data were analyzed using the QIIME 1.5.0 pipeline (Caporaso et al., 2010). Low quality sequences were removed using default parameters. Operational taxonomic units (OTUs) were identified at the 97% identity level. Representative sequences from the OTUs were aligned using PyNAST (DeSantis et al., 2006). The taxonomic affiliation of each OTU was determined using the RDP Classifier at a confidence threshold of 80% against the 12_10 Greengenes core set (Wang et al., 2007). Possible chimeric OTUs were identified using QIIME's ChimeraSlayer and removed from the initially generated OTU list, producing a final set of non-chimeric OTUs.

### Microbiological analyses: statistical analysis

OTU singletons and OTUs related to chloroplasts were removed prior to analyses. The relationship between microbial community composition, diet and time was analyzed by canonical correspondence analysis (CCA) using CANOCO 5 [Ter Braak, C.J.F., Šmilauer, P. *Canoco Reference Manual And User's Guide: Software For Ordination, Version 5.0*. Ithaca: Microcomputer Power, USA. pp. 496 (2012)]. Rare OTUs were down-weighted using the default option. The different diets (p, pB, pE, B, and E) were tested for significant contribution to the explanation of the variation in the OTU distribution with the Monte Carlo permutation test associated with the forward selection subroutine. The OTUs that contributed most to different microbial profiles between diet B and E were calculated using SIMPER in the software package PRIMER 6 v6.1.9 (PRIMER-E Ltd, Plymouth, UK) using normalized OTU tables (square root) of day 14 and day 21.

### mRNAseq: total RNA isolation

GITs of two pools of 10 larvae per diet which had been stored in RNAlater were lysed in QIAzol Lysis Reagent, a Qiagen TissueRuptor was used to cut up the tissue samples and RNA was extracted using the Qiagen miRNeasy Mini Kit according to the manufacturer's description (Qiagen Benelux BV, Venlo, the Netherlands). RNA was eluted in 50 μl and quantified by Nanodrop (Thermo Fisher Scientific, Amsterdam, the Netherlands). Integrity of the RNA was confirmed using an Agilent bioanalyzer2100.

### mRNAseq: library preparation and sequencing

For each sample a RNA-seq library was prepared with the Illumina Truseq mRNASeq Sample Preparation Kit according to the manufacturer's description (Illumina, San Diego CA, USA). Each library was sequenced twice in a paired-end sequencing run with a read length of 50 nucleotides on a Illumina HiSeq2000 with version 2 sequencing chemistry. For each library approximately 20 to 30 million read pairs were obtained.

### mRNAseq: data analysis

Raw reads were quality trimmed using the quality_trim module in the CLCBio assembly cell version 4.01. Reads were mapped to the annotated cDNA's in the ZV9 zebrafish genome assembly using the ref_assembly_short module in the CLCBio assembly cell version 4.01. The data were converted to a table using the assembly_table module in the CLCBio assembly cell version 4.01. A custom perl script was used to convert this table to a tab separated value table. This table was used in R package DESeq v1.0.6 to analyse expression in the different samples (Anders and Huber, 2010). Raw RNA-seq data (reads) have been submitted to the NCBI project data archive under Bioproject number 229446 (Biosample numbers SRS506058 for B1, SRS506087 for B2, SRS506089 for E1, SRS506092 for E2).

Gene expression of differentially expressed genes at *P* < 0.05, both up- or down-regulated, was functionally characterized and classified using DAVID 6.7 (The Database for Annotation, Visualization and Integrated Discovery, Huang et al., 2009a,b).

### Ethics

All experiments were performed in accordance with relevant guidelines and regulations. Protocols used complied with the current laws of the Netherlands and were approved by the Animal Experimental Committee (DEC) of the Wageningen UR in Lelystad (The Netherlands) under number 2011102.

## Results

### Impact of diets on GIT microbial community

The development of the microbial community was assessed over the first 21 days post egg fertilization (dpf). At a confidence threshold of 80%, 97,675 out of 97,894 qualified non-chimeric reads could be assigned to a known phylum using the RDP classifier. Qualified non-chimeric read numbers ranged from 1,740 to 10,685 reads per sample (average: 6,118 reads per sample), and the rarefaction curves showed that samples were sufficiently deep sequenced to discuss similarity and differences for the more abundant OTUs (Figure S1).

All 16S rRNA sequences found in zebrafish 5 dpf were classified as either *Proteobacteria* (48%) or *Firmicutes* (52%). The bacterial composition changed at 7 dpf with the appearance of *Actinobacteria* for both diets tested (paramecium plus diet B: pB, and paramecium plus diet E: pE) and *Bacteroidetes* for zebrafish fed with pE (Figure 1). This was followed by a further increase of diversity at 14 dpf for both diets (B and E without paramecium). The trend of diversifying microbiota was halted at 21 dpf due to the increased dominance of *Proteobacteria*, especially on diet B where they made up for 99% of all reads. Although for day 21, we only had one zebrafish larvae-derived sample per diet, 16S rRNA gene analysis based on RNA-extracted from 4 additional samples (2 for diet B and 2 for diet E) from 21 dpf confirmed the relative abundance of 99% at this day.

**Figure 2:**
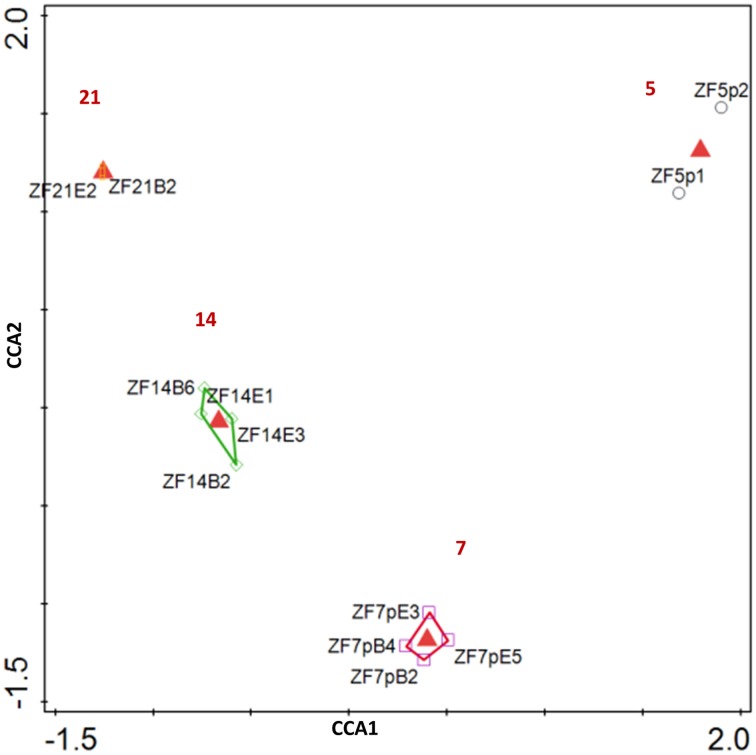
**CCA-ordination plot of the zebrafish microbiota**. The red triangles represent the centroids of the datasets belonging to different time points indicated with the number in red. Each data point refers to DNA extracted from 10 pooled zebrafish. Sample names are build up as follows: *ZF* = zebrafish; dpf (5, 7, 14, or 21); diet (p, pB, pE, B, E); replicate (1, 2).

**Figure 3:**
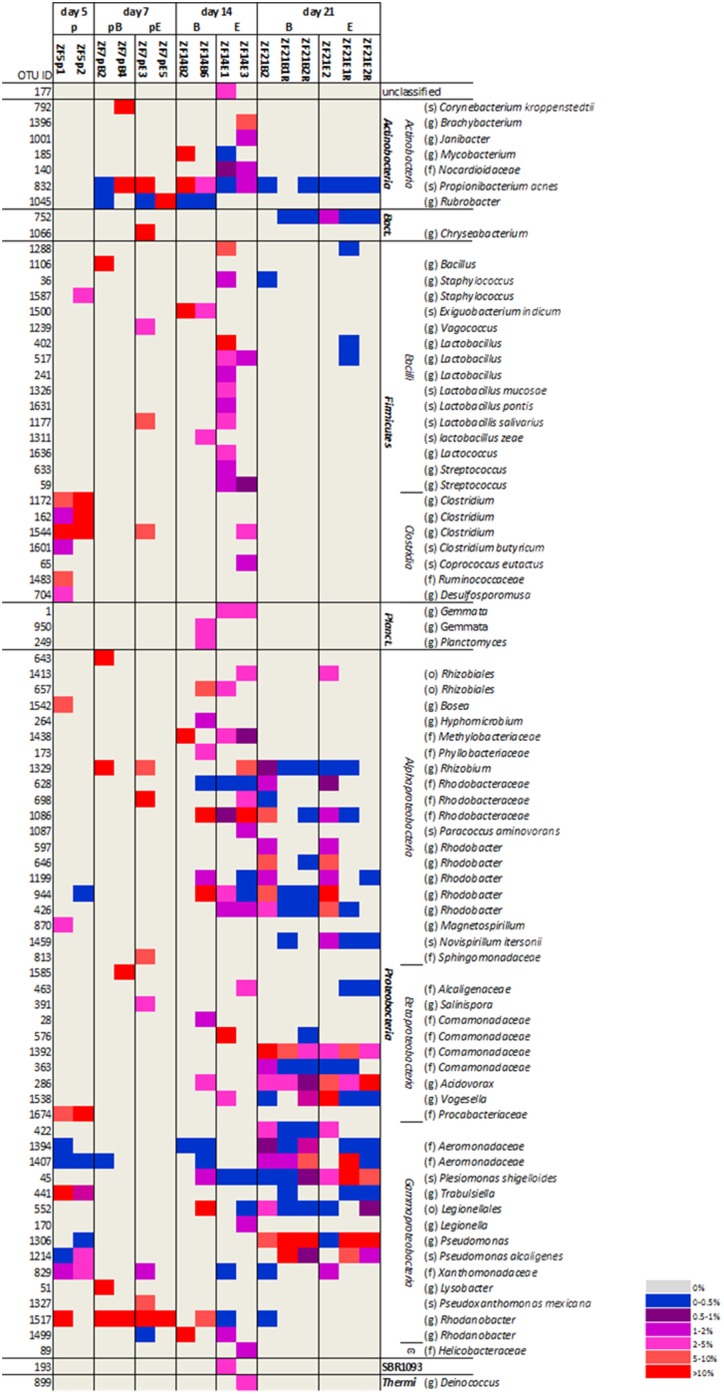
**Heatmap of the operational taxonomic units (OTUs) (97% similarity) that represented more than 1% of the reads in at least one of the zebrafish samples**. These OTUs represented 85–100% of the reads in the different samples. Relative abundance of OTUs is marked according to the legend in the figure. Samples are organized per day and according to diet B or E within day 14 and day 21. OTUs were classified up to the phylum (p), class (c), order (o), family (f), genus (g) or species (s) level. “Bact” refers to the phylum *Bacteroidetes*, “Planct” refers to the phylum *Planctomycetes*. Sample names are built up as follows: *ZF* = zebrafish; dpf (5, 7, 14, or 21); diet (p, pB, pE, B, E); replicate (1, 2); (R) if a sample is derived from RNA.

The bacterial colonization of zebrafish and the impact of diet on the colonization were analyzed at the approximate species level (97% identity based on rRNA gene sequence) by canonical correspondence analysis (CCA). The first two CCA axes had eigenvalues of 0.89 and 0.80, respectively and explained 27% of the variation in species data and 72% of the variation in the microbial taxa-time interactions (Figure 2). The microbiota changed dramatically during the first 21 dpf and the high impact of time (*P* = 0.002) masked treatment (diet) effects. Therefore, the impact of diet on zebrafish-associated microbiota was based on the zebrafish gut-derived RNA samples on day 21 for diet B and diet E for which replicate samples were available. These RNA-based samples were similar to the DNA-based samples for 21 dpf with respect to their microbial profiles at the OTU-level (Figure S2), which indicates that the approach used to obtain 16S rRNA gene amplicons (whole fish + DNA extraction vs. fish gut + RNA extraction and reverse transcription) did not have a major impact on the data obtained. The dominant early bacterial colonizers of zebrafish included members of the *Clostridia* (*Firmicutes*) and *Procabacteriaceae, Trabulsiella* and *Xanthomonadaceae* (all *Proteobacteria*) (Figure 3). These OTUs were mostly absent at day 7 and were replaced by OTUs most closely related to *Propionibacterium acnes* (*Actinobacteria*), *Rhodanobacter*, and *Rhizobium* (both *Proteobacteria*). *Propionibacterium acnes* was still highly abundant at day 14 as was *Rhodanobacter* albeit at a lower percentage. Populations within the *Rhodobacteraceae* and *Methylobacteraceae* (both *Proteobacteria*) were newly appearing dominant OTUs at day 14. Although OTUs most closely related to *Propionibacterium acnes* and *Rhodobacteraceae* were still found at low levels at day 21, those falling within *Rhodanobacter* and *Methylobacteraceae* had disappeared. Instead, populations within the *Comamonadaceae, Aeromonadaceae, Acidovorax*, and *Pseudomonas* (all *Proteobacteria*) dominated the zebrafish-associated microbiota.

The OTUs that contributed most (1% or more) to the differences in microbial profiles in zebrafish fed with diet B or diet E at day 21 were identified by SIMPER (Table 2). OTUs with a higher relative abundance for diet E that mainly contributed to the differences in microbial profiles between the diets were OTU45 (*Plesiomonas shigelloides*), OTU286 (*Acidovorax* sp.), OTU1407 (family *Aeromonadaceae*), OTU 441 (*Trabulsiella* sp.), OTU552 (order *Legionellales*), OTU1459 (*Novospirillum itersonii*), OTU1414 (*Rheinheimera* sp.), OTU832 (*Propionibacterium acnes*), OTU463 (family *Alcaligenaceae*), OTU16 (*Burkholderia* sp.), and OTU 1182 (*Halomonas* sp.). Other OTUs that contributed less to the difference observed between diet E and B, but which were completely absent in zebrafish fed with diet B were OTU1278 (*Achromobacter* sp.), OTU568 (*Janibacter* sp.), and OTU1260 (*Cupriavidus* sp.). The OTUs with a higher relative abundance with diet B were *Pseudomonas* spp. (OTU1214 and OTU1306), which were among the most dominant OTUs found in zebrafish at day 21 and had an even higher relative abundance for diet B than for diet E.

**Table 2:**
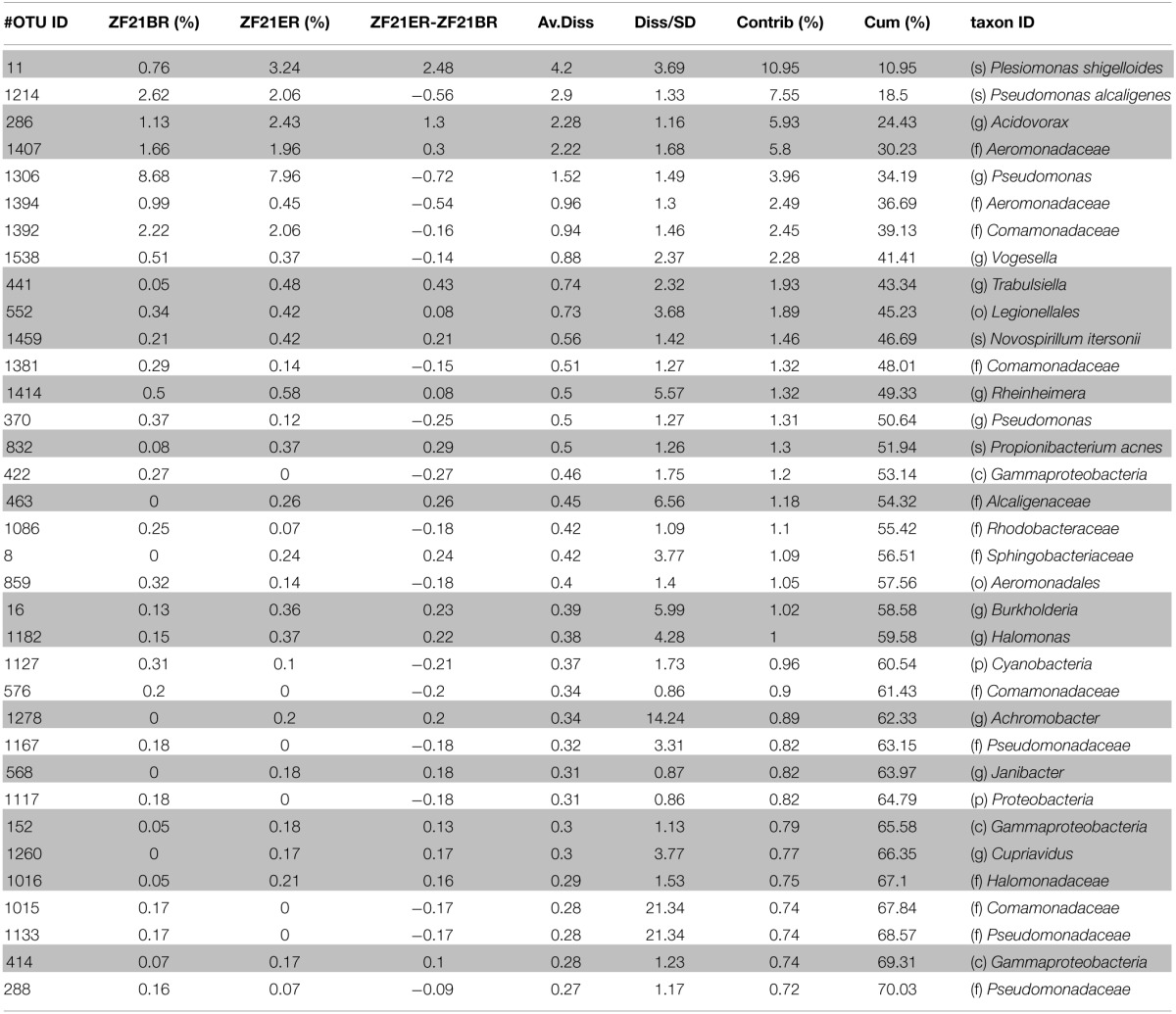
**Bacterial OTUs that contribute most to the difference between diet E and diet B at day 21**.

*Only OTUs that cumulatively contribute to 50% of the difference between bacterial profiles between diet B and E are shown based on SIMPER analysis. Average relative abundances of square root-transformed data for diet B and E is indicated in the columns ZF21B and ZF21E, respectively. “Av.Diss.” indicates the average dissimilarity between the diets for each OTU; “Diss/SD” indicates the dissimilarity divided by the standard deviation; “Contrib” is the relative contribution to the difference between diet B and E; and “Cum” represents the cumulative relative contribution to the difference starting from the top with the OTUs that contribute most to the difference. OTUs in gray refer to OTUs that were more abundant for diet E. OTUs were classified up to the phylum (p), class (c), order (o), family (f), genus (g) or species (s) level*.

### mRNAseq gene expression analyses

In order to assess potential differences in how the zebrafish host responds to the different dietary treatments, intestinal tissue-associated gene expression was measured at 21 dpf. Reads were mapped to the 27,882 annotated cDNA's of the ZV9 zebrafish genome assembly that were used for further analysis. In total, 328 genes were differentially expressed corresponding to 1.18% of the total number of genes (Table S1). Of these, expression of 214 genes was up-regulated and expression of 114 genes was down-regulated in larvae fed with diet E vs B. Among these, expression of 16 differentially expressed genes were detected exclusively for larvae fed with diet E and were below detection thresholds for larvae of diet B (fold change—fc “inf”), whereas for 10 other differentially expressed genes the opposite was true (fc “0”).

Of the total number of 27,882 genes that were analyzed, 19,990 genes could be converted to a DAVID id, and of the total number of 328 differentially regulated genes, 264 genes could be converted as such. Genes with a DAVID id were used for unbiased functional annotation.

Functional annotation clustering revealed that the differentially expressed genes represented 27 annotation clusters. 24 terms representing 7 of these clusters were significantly enriched at *P* < 0.05 (Table S2). 52 terms were not clustered. The functional annotation clusters were associated with *ribosome components and activity* (enrichment score—es 14.61) and *transport* (es 1.4) as the dominant clusters. Other clusters involved *other glycan degradation* (es 1.25); *glycosaminoglycan metabolic process* (es 1.13); *extracellular matrix structural constituent* (es 0.66); *keratin type-1* (es 0.61) and *hydrogen ion transmembrane transporter activity* (es 0.60). The functional annotation chart revealed 65 records of which 40 were significantly enriched (Table S3). In addition to many terms that were associated with *ribosome, transport* and the other mentioned clusters, enriched records were associated with: *interferon-induced 6-16, interferon binding* and *interferon receptor activity*; *glycoside hydrolase* and *oxidative phosphorylation*.

Gene functional classification showed the presence of 7 gene groups as determined by 73 differentially expressed genes (Table 3, Table S4) agreeing with the existence of 7 enriched functionally annotated clusters. Also here the dominant gene groups represented *ribosome components and activity* and *transport*. The gene group *ribosome components and activity* consisted of 27 genes that were all significantly up-regulated at fc 1.47–1.85 in larvae fed with diet E vs. those fed with diet B (75 vs. 10% NPM). Also in the gene group *transport* all four genes were up-regulated at fc 1.56 up to 24.20 for *solute carrier family 12 (sodium/chloride transporters), member 3*. Other groups involved *WD40 repeats acting as protein-protein interaction sites*; *nucleotide binding*; *transcription*; *metal binding/zinc fingers* and *membrane*. In gene groups *WD40 repeats* (4 genes) and *metal binding/zinc fingers* (13 genes) all genes were up-regulated while groups *nucleotide binding, transcription* and *membrane* contained both genes that were up-regulated as well as genes that were down-regulated. Among them were genes that were specific for larvae fed with diet E (*zgc:110560* or *hypothetical protein LOC100150958*; *similar to Serine/threonine-protein kinase Pim-3* or *si:dkey-108d22.5*; *forkhead box G1* and *mediator of RNA polymerase II transcription subunit 11*) and genes specific for larvae fed with diet B (*zgc:172065* or *hypothetical LOC100001153*).

**Table 3:**
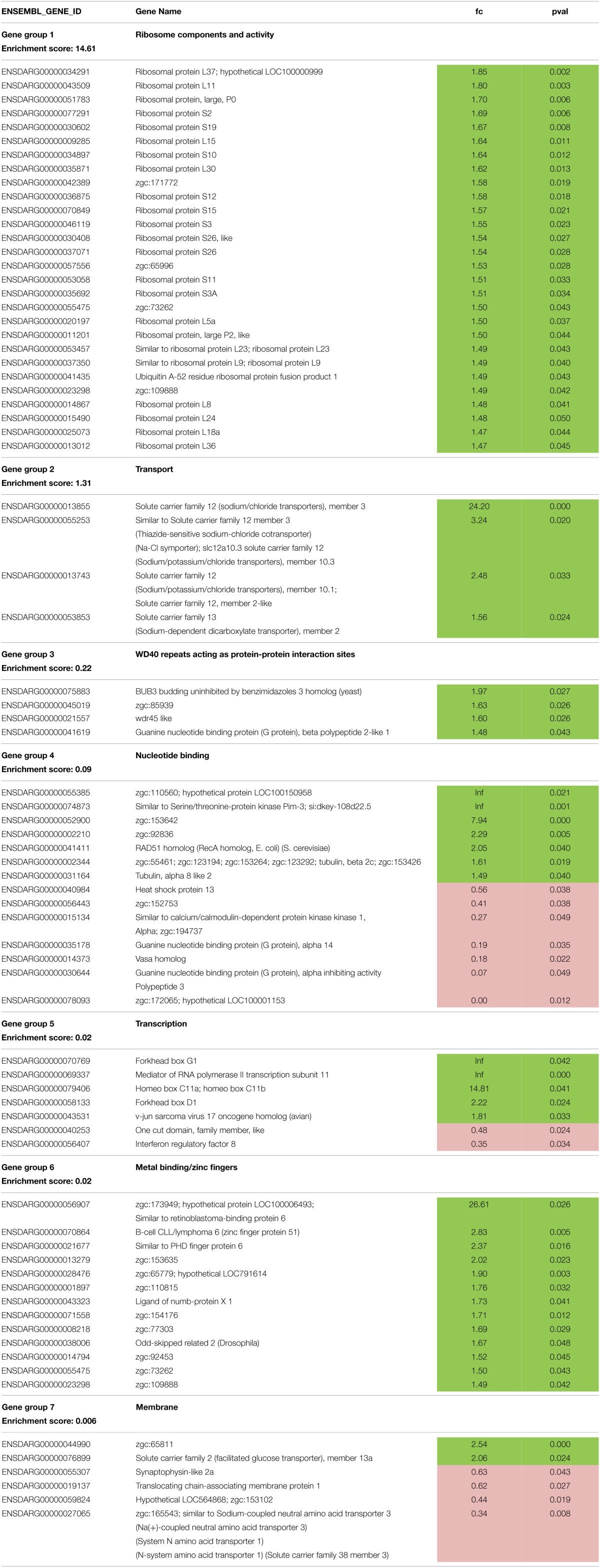
**Functional gene groups and their differentially expressed genes**.

*Shown are each of the functional gene groups, enrichment score and name; the Ensemble gene ID and gene name; fold change and P-values for differentially expressed genes (P ≤ 0.05)*.

*Up-regulated gene expression is marked green and down-regulated gene expression is marked red*.

## Discussion

This study represents a methodological exploration assessing the development of the gastrointestinal microbiota through 16S rRNA gene-based microbial composition profiling by pyrosequencing and the functional response of the transcriptome by mRNAseq. Both approaches provided complementary information on the nutritional impact of a novel protein source relevant for aquaculture. The impact of the novel protein meal concerned the impact on the GIT microbiota and, either directly or indirectly through the microbiota, on the host transcriptome.

The two experimental diets especially differed in the level of iron (Table 1). This difference originated from the difference in NPM as it is a rich iron source, consisting mostly of heme iron. Feed ingredients of animal origin, like the NPM in this study, are rich in iron. Iron can be taken up in two forms; as heme (e.g., hemoglobin), and as non-heme (e.g., iron sulfate). The uptake of heme differs greatly from the absorption of inorganic iron as the uptake of heme iron is not physiologically regulated and independent of the intestinal pH in contrast to the uptake of inorganic iron (Kraemer and Zimmermann, 2007). Uptake of inorganic iron is more complex and requires reduction of Fe^3+^ to Fe^2+^ which, in turn, requires an acidic environment as provided by the gut in monogastric animals. Despite very few mechanistic studies of piscine intestinal iron uptake, zebrafish is supposed to take up iron from the diet in the intestinal enterocytes not any different than by the mechanism that all vertebrates apply (reviewed by Bury et al., 2003). Iron homeostasis is crucial since, in excess, iron can be detrimental to health because of its production of oxygen free radicals, and, when too low, loss of energy due to the decrease of hemoglobin concentrations and cytochrome capacity in aerobic metabolism. In diets B and E, calculated iron content was 312 and 1486 mg kg^−1^, respectively (Table 1). These levels exceed the daily iron requirements in fish that ranges between 30 and 170 mg kg^−1^ DM food (Watanabe et al., 1997). Also experimental studies on dietary iron supplementation in fish report on findings that indicate that such levels have already reached a plateau for physiological effects (tilapia: Shiau and Su, 2003; rainbow trout: Carriquiriborde et al., 2004). So most probably any difference in GIT response to the experimental diets does not relate to an iron shortage in fish fed with diet B but may reflect effects of iron overloading, especially in fish fed with diet E.

Of 97,894 representative sequences of GIT microbiota in the present study, the dominant phylum was *Proteobacteria*. Other main phyla encountered and ranked according to average relative abundance were *Firmicutes, Actinobacteria*, and *Bacteroidetes*. The rapid temporal changes in GIT microbiota make it difficult to compare our results directly to other studies that have been done on the identification of zebrafish GIT microbiota (Bates et al., 2006; Roeselers et al., 2011; Lan and Love, 2012; Semanova et al., 2012). However, our data are similar to published data for two important aspects: (i) larval zebrafish GITs are dominated by one to three bacterial species, (ii) these dominant species generally belong to *Proteobacteria* and *Firmicutes*.

Firstly, many larval and juvenile animals still have immature gut microbiota that are not yet fully functional and may be dominated by a few early colonizers. For human gut microbiota it has been shown that infant gut bacterial species have faster growth rates than adult gut bacterial species, which favors early colonization (De Muinck et al., 2013). With respect to the second point, dominant bacteria in the GITs of fish juveniles have been identified as *Pseudomonas* (*Proteobacteria*) in zebrafish and salmon (Bates et al., 2006; Navarette et al., 2009), and an unidentified gammaproteobacterium in juvenile pinfish (Givens, 2012). Similarly, the GIT microbiota of juvenile *Siberian sturgeon* was shown to be mono-dominated, only by *Cetobacterium somerae* (*Fusobacteria*) (Geraylou et al., 2013). Thereby it should be noted that the herbivorous pinfish and the carnivorous salmon and sturgeon have stomachs and GIT morphologies that are different from the omnivorous stomachless zebrafish.

At 5 dpf, zebrafish GIT in our study was dominated by members of the genus *Clostridium* (*Firmicutes*) with 40 ± 11% of the reads and the family *Procabacteriaceae* (*Proteobacteria*) with 22 ± 21% of the reads. *Clostridium* is a well-known inhabitant of the animal gut. Although the genus *Clostridium* has been related to animal diseases, commensal *Clostridium* spp. are dominant players in the maintenance of gut homeostasis in man and other animals including many fish species (Sullam et al., 2012, Lopetuso et al., 2013). *Clostridium* spp. were previously found to be abundant in the GIT of fed juvenile zebrafish (Semanova et al., 2012), but absent or not abundant in starved juveniles and in the adult zebrafish GIT (Roeselers et al., 2011; Semanova et al., 2012), which may indicate that it is an early colonizer of the zebrafish GIT that disappears with gut maturation. This is also confirmed by our data as *Clostridium* spp. were detected only in a few samples at later time points. Little information exists on the family *Procabacteriaceae* (*Proteobacteria*) and they are not frequently encountered in animal gut. Candidatus *Procabacter acanthamoebae* was identified as an *Acanthamoeba* endosymbiont (Horn et al., 2002). *Acanthamoeba* is related to a number of animal diseases (Paterson et al., 2011), however, no visible signs of distress were recorded at 5 dpf and onwards no more reads were detected that were affiliated to *Procabacteriaceae*. The zebrafish-associated microbiota shifted remarkably between 5 and 7 dpf (Figure 2) and only few of the OTUs present at 5 dpf were also found at 7 dpf. One OTU that was recovered at both days was a *Rhodanobacter* sp. (*Proteobacteria*) that accounted for 37 ± 34% of the reads at 7 dpf. *Rhodanobacter* spp. are typically known for their potential for partial or complete denitrification (Kostka et al., 2012) and may have been derived from the fish tank water filtration system. The other dominant OTU at 7 dpf was closely related to *Propionibacterium acnes* (*Actinobacteria*) and this OTU remained traceable at 14 and 21 dpf albeit at lower relative abundance. A large number of new OTUs was found at 14 dpf of which many were lost again at 21 dpf. However, some, such as a number of OTUs belonging to the family *Rhodobacteraceae*, appeared at 14 dpf and remained. *Rhodobacteraceae* are commonly found in the aquatic habitat, but are not typical gut-associated bacteria (Elifantz et al., 2013). Despite the large fluctuations of zebrafish-associated microbiota over time, it is apparent that the inter-individual variation within time points decreases (Figure 2, Figure S2), which indicates that a more stable and homogenous microbiota becomes associated with the zebrafish population at 21 dpf. In addition, the dominant OTU at 21 dpf, a *Pseudomonas* sp., is in line with previous studies in zebrafish (Bates et al., 2006; Roeselers et al., 2011; Lan and Love, 2012; Semanova et al., 2012), which could indicate gut maturation.

The OTUs that were present at a higher relative abundance in fish fed with the experimental diet E as compared to animals fed with the control diet B and contributed most to the difference between gut microbiota for the different diets can roughly be divided into three groups based on comparison to their near neighbors: (1) potential pathogens, (2) bacteria with an increased iron metabolism, and (3) common aquatic bacteria. From the first group, *Plesiomonas shigelloides* (OTU45) contributed most to the difference in microbiota between diet E and diet B. It is an emerging pathogen that is widespread in the aquatic environment and has been related to gastrointestinal infections and other diseases in a wide range of animal hosts including fish (Chen et al., 2013; Joh et al., 2013). Also the family *Aeromonadaceae* (to which OTU1407 belongs) harbors many fish pathogens that are associated to gastroenteritis and wound infections (Tomás, 2012). The genus *Trabulsiella* was proposed in 1991 (McWhorter et al., 1991) as a genus that is highly related to pathogenic *Salmonella* sp. Currently two species belonging to this genus have been described and were isolated from human and termite gut. Although *T. guamensis* can occur in human diarrheal stools, there still is no evidence that it actually causes diarrhea (McWhorter et al., 1991; Chou et al., 2007). The order *Legionellales* (OTU 552) comprises the families *Legionellaceae* and *Coxiellaceae* that are both known to represent common animal pathogens (Garrity et al., 2005). The genus *Burkholderia* (OTU16) represents both pathogenic (animals and plants) and non-pathogenic species (Estrada-de los Santos et al., 2013). *Propionibacterium acnes* (OTU832) and *Cupriavidus* (OTU1260) spp. are commensal inhabitants of the skin and GIT of animals, but are also related to infections, especially in immuno-compromised individuals (Perry and Lambert, 2006; Balada-Llasat et al., 2010). In addition, some *Cupriavidus* spp., such as *C. gilardii* and *C. metallidurans* are particularly resistant to high metal concentrations (Kirsten et al., 2011). The *Halomonas* sp. (OTU1182) that was markedly increased in zebrafish fed at high NPM inclusion level shared 96–98% percent identity with *Halomonas titanicae* strains SSA831, SSA728 and SSA637 based on the 16S rRNA gene. *Halomonas titanicae* was isolated from corroded parts of the RMS Titanic wreck and possesses an unusual high number of iron reductases, iron uptake regulators, ferrochelase, iron transporters, and iron-binding periplasmic protein-encoding genes (Sánchez-Porro et al., 2013). In addition, also *Novospirillum itersonii* (OTU1459), previously named *Aquaspirillum itersonii*, is best known for its iron reduction capacities (Dailey and Lascelles, 1977) and may have been selected for by the high iron content of diet E. In future studies, a tank water control should be included, in this case to be able to confirm that these iron reducers originated from the tank water.

In this study, we have applied mRNAseq in an unbiased approach to investigate the molecular differentiation of physiological processes in the GIT as indicated by differentially expressed genes. In other recent studies that we found in literature, only whole-body mRNAseq was performed at such young stages of developing zebrafish, or microarray studies specifically on the GIT. Note that because in this study the whole GIT was analyzed, any variation in physiological processes occurring along the GIT was thereby discarded (Clements et al., 2014). Although zebrafish belongs to the cyprinid family, a family of fishes that that do not possess a stomach, also stomachless fish show regional differentiation in GIT function (German, 2009) and microbial communities (Clements et al., 2014). In our study only approximately 1% of the total number of genes was differentially expressed. Functional classification of genes revealed that by far the most dominant gene groups represented *ribosome components and activity* and *transport* that were enriched in their expression in the larvae fed at high inclusion levels vs. those fed at low inclusion levels of the NPM. These gene groups included 23 ribosomal proteins and several solute carrier families of sodium, potassium, chloride, dicarboxylate, and aminoacid and glucose transporters. These data would suggest that the GIT of larvae fed at higher inclusion of the protein meal is much more active in transmembrane ion transport and protein synthesis, perhaps for making the machinery to perform this transport.

Among the individual genes, we have found 10 uniquely expressed genes for fish fed at low inclusion levels and 16 genes for fish fed at high inclusion levels. The 10 uniquely expressed genes for fish fed at low inclusion levels were all uncharacterised genes except for one: *secretogranin V (7B2 protein)*, a gene required for the production of an active Proprotein convertase 2 (PC2) enzyme (also known as prohormone convertase 2 or neuroendocrine convertase 2 enzyme) that is responsible for the first step in the maturation of many neuroendocrine peptides from their precursors, such as the conversion of proinsulin to insulin intermediates (Mbikay et al., 2001; Portela-Gomes et al., 2008). The 16 uniquely expressed genes for fish fed at high inclusion levels were all, except for four genes, characterized. Among them were *cadherin 16, KSP-cadherin*, a calcium-dependent, membrane-associated glycoprotein, and *claudin 19*, involved in magnesium transport. These genes also have a clear role in *transport* and so has the highest up-regulated expressed gene in fish fed at high inclusion level at fc 768: *stanniocalcin 1, like*. Stanniocalcin 1 is involved in calcium homeostasis. It has been found to reduce Ca^2+^ uptake via the inhibition of epithelial Ca^2+^ channel mRNA expression in zebrafish embryos (Tseng et al., 2009). As such it would fit well with a role in the GIT, with the dominant gene group *transport* and with other strongly up-regulated genes expressing channels and transporters (*purinergic receptor P2X, ligand-gated ion channel, 3b* at fc 102; *solute carrier family 12 (sodium/chloride transporters), member 3* at fc 24; *chloride channel accessory 2* at fc 4.93; *slc12a10.3 solute carrier family 12 (sodium/potassium/chloride transporters), member 10.3* at fc 3.24; *solute carrier family 16 (monocarboxylic acid transporters), member 9a* at fc 2.86; *solute carrier family 12, member 10.1* at fc 2.48; *solute carrier family 25, member 38a* at fc 2.06; *solute carrier family 2 (facilitated glucose transporter), member 13* at fc 2.06). However, such high level of difference in expression is often indicative for immune-related genes. Stanniocalcin 1 also has an immune-related function. It is an inhibitor of macrophage chemotaxis and chemokinesis (Kanellis et al., 2004) and modulates transendothelial migration of leukocytes (Chakraborty et al., 2007) in humans. Thus, a role in modulating the immune/inflammatory response could be expected. There are more signs for an immune response in fish fed at high NPM levels given the roles of other up-regulated immune-related genes such as *interlectin 2* at fc 8.64; *radical S-adenosyl methionine domain containing 2* at fc 6.29 (see also later); *ISG15 ubiquitin-like modifier* at fc 4.99 and *B-cell CLL/lymphoma 6a (zinc finger protein 51)* at fc 2.83. Rawls et al. (2004) performed DNA microarray comparisons of gene expression in the digestive tracts of 6 dpf zebrafish and revealed that 212 genes were regulated by the microbiota, including genes involved in innate immune responses. Thus, we cannot rule out that fish fed at high NPM levels display an gastrointestinal immune response.

Because the two experimental diets especially differed in the level of iron, in a biased approach, we have analyzed the expression profiles of genes functionally involved in iron uptake and homeostasis. Twenty seven genes were identified as involved in iron homeostasis but were non-differentially expressed at a fold change 0.27–1.54 (Table S5). Among them were genes encoding for *ferritin, transferrin receptors, hephaestin, ferrochelatase*, an *iron-responsive element binding protein*, an *iron-regulated transporter* and *ceruloplasmin*; but also *heme oxygenase, heme binding protein*, a *heme transporter*, and finally *hepcidin* and the *interleukin 6 receptor*. In a parallel study we have investigated the effects on adult zebrafish fed with the same experimental diets for 1 month (Palstra et al., unpublished data). In a biased approach, quantitative real-time PCR was performed on individual GIT and liver of these fish. Here we did find significant differential expression of several of these genes: GIT expression of marker gene *hepcidin antimicrobial peptide 1* (*hamp1*) was significantly higher, and of *hephaestin-like 1* significantly lower in fish fed at high vs. low iron level. Liver expression of marker genes *transferrin a* and *hamp1* was significantly higher, and of *ferritin heavy polypeptide 1a* significantly lower in fish fed at high vs. low iron level. These expression profiles, supported by data on body composition, suggest that in adult fish fed at higher iron level, less dietary iron uptake occurs, less iron is released in the circulation, and less iron is taken up and stored in the liver. This suggests a metabolic defense mechanism against iron overload. Indeed, metal absorption is lower when metal concentrations are elevated (reviewed by Karasov and Douglas, 2013). However, as based on the absence of differential expression of such genes for the larval zebrafish in this study, we cannot conclude that major changes occur in iron uptake and homeostasis during the earliest stages of development. Although diets especially differed in iron content, no data have been collected in this study that show that this difference also leads to a difference in iron availability to the GIT. The competitory activity of the microbiota may result in an alteration of the iron availability for the gastrointestinal functions. Some genes that were differentially expressed may have a relation with iron homeostasis. Among them was *wdr45 like* that was up-regulated at fold change 1.6 in larvae fed at higher iron level. Wdr45 is associated with human brain iron accumulation (Haack et al., 2012). *Radical S-adenosyl methionine domain containing 2* (*rsad2*) is an interferon-inducible iron-sulfur cluster-binding antiviral protein that was up-regulated at fold change 6.29 at higher iron levels. Other genes that may be involved could be many of the unknown differentially expressed genes belonging to the cluster metal binding/zinc fingers.

The developing gastrointestinal microbiota of larval zebrafish showed rapid temporal changes until a suspected stable and mature state at 21 dpf. At all times, the larval zebrafish GITs were dominated by one to three bacterial species generally belonging to *Proteobacteria* and *Firmicutes*. The OTUs that contributed most to the difference between gastrointestinal microbiota for the different diets represented common aquatic bacteria but also bacteria related to potential pathogens and bacteria with an increased iron metabolism. As for the gastrointestinal transcriptome at 21 dpf, the GIT of larvae fed at higher NPM inclusion is more active in transmembrane ion transport and protein synthesis. Although some indications existed, transcriptomic analysis did not reveal signs for the occurrence of a major immune/inflammatory activation and/or iron overload response.

The gained insights on the impact of the NPM on larval zebrafish GIT microbiology and physiology are valuable information for fish specifically, and vertebrates in general. Caution is required with the transfer of knowledge toward commercially produced species in aquaculture, particularly because many of them are carnivorous. Carnivorous fishes like salmon (Navarette et al., 2009) and sole (Martin-Antonio et al., 2007; Tapia-Paniagua et al., 2010) possess a specialized GIT region with an acidic environment, or a stomach, while the omnivorous cyprinid zebrafish is stomachless. Such interspecific differences in GIT morphology have important consequences for the GIT microbial composition and physiology (Clements et al., 2014), and thus for the dietary impact of the NPM.

## Author contributions

Conceived and designed the experiments: ER, DS, JK, AP. Performed the experiments: ER, DS, MV, MF, GB. Analyzed the data: ER, DS, AP. Wrote the paper: ER, DS, JK, HS, AP.

### Conflict of interest statement

The authors declare that the research was conducted in the absence of any commercial or financial relationships that could be construed as a potential conflict of interest.
