# Supplementary material for: Impact of a novel protein meal on the gastrointestinal microbiota and the host transcriptome of larval zebrafish Danio rerio
Source: Front Physiol. 2015 Apr 30;6:133. doi: 10.3389/fphys.2015.00133 (PMC4415425; doi:10.3389/fphys.2015.00133)
Supplement: Supplementary file 1 [file DataSheet1.DOCX]

***Supplementary Material***

**Impact of a novel protein meal on the gastrointestinal microbiota and transcriptome of larval zebrafish *Danio rerio***

**Eugene Rurangwa^1#^, Detmer Sipkema^2#^, Jeroen Kals^1^, Menno ter Veld^3^, Maria Forlenza^4^, Gianina M. Bacanu^2^, Hauke Smidt^2^, Arjan P. Palstra^1^***

^1^ Institute for Marine Resources and Ecosystem Studies (IMARES), Wageningen University and Research Centre, Yerseke, the Netherlands

^2^ Laboratory of Microbiology, Wageningen University, Wageningen, the Netherlands

^3^ Aquaculture and Fisheries Group, Wageningen University, Wageningen, the Netherlands

^4^ Cell Biology and Immunology Group, Wageningen University, Wageningen, the Netherlands

*** Correspondence:** Dr. Arjan P. Palstra, The Institute for Marine Resources and Ecosystem Studies (IMARES), Wageningen University and Research Centre, Korringaweg 5, 4401 NT Yerseke, The Netherlands

[arjan.palstra@wur.nl](mailto:arjan.palstra@wur.nl)

1. **Supplementary Data**
2. **Supplementary Figures and Tables**

## Supplementary Tables

**Table S1. Differentially expressed genes.**

Shown are the ensemble gene id, gene description, fold change and P-value of all 328 differentially expressed genes (P≤0.05). Expression of 16 differentially expressed genes could only be detected for larvae of diet E and were below detection thresholds for larvae of diet B (fold change - fc “inf”) and for 10 differentially expressed genes the opposite was true (fc “0”).

| **id** | **gene** | **fc** | **pval** |
| --- | --- | --- | --- |
| ENSDART00000032967 | notch homolog 2 [Source:RefSeq peptide;Acc:NP_001108566] | Inf | 3.26E-32 |
| ENSDART00000122571 | cyclin-dependent kinase inhibitor 1B (p27, Kip1) [Source:HGNC Symbol;Acc:1785] | Inf | 2.51E-22 |
| ENSDART00000073736 | reticulon 4 receptor-like 2 a [Source:ZFIN;Acc:ZDB-GENE-040310-4] | Inf | 8.64E-07 |
| ENSDART00000127250 | serine/threonine kinase 35 [Source:HGNC Symbol;Acc:16254] | Inf | 2.94E-06 |
| ENSDART00000027000 | rhodopsin [Source:ZFIN;Acc:ZDB-GENE-990415-271] | Inf | 7.14E-06 |
| ENSDART00000109534 | cadherin 16, KSP-cadherin [Source:HGNC Symbol;Acc:1755] | Inf | 2.28E-04 |
| ENSDART00000066154 | mediator complex subunit 11 [Source:HGNC Symbol;Acc:32687] | Inf | 3.95E-04 |
| ENSDART00000113595 | Uncharacterized protein [Source:UniProtKB/TrEMBL;Acc:F1RD44] | Inf | 1.05E-03 |
| ENSDART00000073378 | Uncharacterized protein [Source:UniProtKB/TrEMBL;Acc:F1RAH9] | Inf | 3.01E-03 |
| ENSDART00000143866 | si:ch211-223o1.8, pseudogene [Source:ZFIN;Acc:ZDB-GENEP-050419-1] | Inf | 9.19E-03 |
| ENSDART00000131265 | si:dkey-241l7.10 [Source:ZFIN;Acc:ZDB-GENE-041014-236] | Inf | 1.12E-02 |
| ENSDART00000075568 | noggin [Source:HGNC Symbol;Acc:7866] | Inf | 1.25E-02 |
| ENSDART00000077728 | PAP associated domain containing 4 [Source:ZFIN;Acc:ZDB-GENE-050522-536] | Inf | 2.09E-02 |
| ENSDART00000059308 | SPC24, NDC80 kinetochore complex component, homolog (S. cerevisiae) [Source:ZFIN;Acc:ZDB-GENE-050522-456] | Inf | 2.52E-02 |
| ENSDART00000104204 | forkhead box G1a [Source:ZFIN;Acc:ZDB-GENE-990415-267] | Inf | 4.18E-02 |
| ENSDART00000065461 | claudin 19 [Source:ZFIN;Acc:ZDB-GENE-050417-242] | Inf | 4.89E-02 |
| ENSDART00000081347 | stanniocalcin 1, like [Source:ZFIN;Acc:ZDB-GENE-040426-1521] | 768.25 | 6.17E-98 |
| ENSDART00000133926 | purinergic receptor P2X, ligand-gated ion channel, 3b [Source:ZFIN;Acc:ZDB-GENE-030319-3] | 101.87 | 1.75E-03 |
| ENSDART00000114502 | si:ch211-22d5.2 [Source:ZFIN;Acc:ZDB-GENE-091204-283] | 66.15 | 4.53E-02 |
| ENSDART00000139640 | si:rp71-45g20.10 [Source:ZFIN;Acc:ZDB-GENE-070912-707] | 26.61 | 2.61E-02 |
| ENSDART00000010998 | solute carrier family 12 (sodium/chloride transporters), member 3 [Source:ZFIN;Acc:ZDB-GENE-030131-9505] | 24.20 | 2.51E-26 |
| ENSDART00000133193 | si:dkey-224k5.10 [Source:ZFIN;Acc:ZDB-GENE-060531-110] | 18.86 | 4.90E-03 |
| ENSDART00000145960 | zgc:163136 [Source:ZFIN;Acc:ZDB-GENE-070615-14] | 17.95 | 8.71E-03 |
| ENSDART00000111698 | homeo box C11b [Source:ZFIN;Acc:ZDB-GENE-000822-3] | 14.81 | 4.09E-02 |
| ENSDART00000074900 | hypothetical protein LOC567953 [Source:RefSeq peptide;Acc:NP_001122213] | 13.32 | 3.11E-02 |
| ENSDART00000100100 | adrenomedullin [Source:HGNC Symbol;Acc:259] | 9.76 | 1.39E-02 |
| ENSDART00000124878 | Uncharacterized protein [Source:UniProtKB/TrEMBL;Acc:F1QCY3] | 9.56 | 3.24E-02 |
| ENSDART00000122617 | Uncharacterized protein [Source:UniProtKB/TrEMBL;Acc:E7EYP1] | 9.50 | 2.68E-04 |
| ENSDART00000139345 | si:dkey-14d8.12 [Source:ZFIN;Acc:ZDB-GENE-041210-135] | 8.79 | 6.79E-04 |
| ENSDART00000073554 | intelectin 2 [Source:ZFIN;Acc:ZDB-GENE-050411-58] | 8.64 | 1.06E-06 |
| ENSDART00000005485 | zgc:136902 [Source:ZFIN;Acc:ZDB-GENE-060421-4592] | 7.97 | 5.23E-06 |
| ENSDART00000093269 | zgc:153642 [Source:ZFIN;Acc:ZDB-GENE-061013-527] | 7.94 | 1.42E-05 |
| ENSDART00000019617 | radical S-adenosyl methionine domain containing 2 [Source:ZFIN;Acc:ZDB-GENE-050913-129] | 6.29 | 3.57E-02 |
| ENSDART00000045967 | cytidine monophosphate (UMP-CMP) kinase 2, mitochondrial [Source:ZFIN;Acc:ZDB-GENE-040724-24] | 5.04 | 2.42E-02 |
| ENSDART00000130554 | ISG15 ubiquitin-like modifier [Source:ZFIN;Acc:ZDB-GENE-021211-1] | 4.99 | 5.46E-03 |
| ENSDART00000130524 | chloride channel accessory 2 [Source:HGNC Symbol;Acc:2016] | 4.93 | 2.77E-06 |
| ENSDART00000043961 | myeloid-specific peroxidase [Source:ZFIN;Acc:ZDB-GENE-030131-9460] | 4.73 | 2.63E-08 |
| ENSDART00000111547 | si:dkeyp-118h9.7 [Source:ZFIN;Acc:ZDB-GENE-091204-477] | 4.57 | 4.17E-02 |
| ENSDART00000074720 | discs, large homolog 3 (Drosophila) [Source:HGNC Symbol;Acc:2902] | 3.71 | 2.31E-03 |
| ENSDART00000018064 | zgc:152791 [Source:ZFIN;Acc:ZDB-GENE-060901-4] | 3.59 | 1.39E-02 |
| ENSDART00000084689 | Uncharacterized protein [Source:UniProtKB/TrEMBL;Acc:F1R1D7] | 3.36 | 2.77E-04 |
| ENSDART00000003347 | ring finger protein 213 [Source:HGNC Symbol;Acc:14539] | 3.27 | 1.26E-02 |
| ENSDART00000042955 | slc12a10.3 solute carrier family 12 (sodium/potassium/chloride transporters), member 10.3 [Source:ZFIN;Acc:ZDB-GENE-060503-518] | 3.24 | 2.03E-02 |
| ENSDART00000105036 | type I cytokeratin, enveloping layer, like [Source:ZFIN;Acc:ZDB-GENE-061026-4] | 3.22 | 1.04E-02 |
| ENSDART00000125862 | si:ch211-171l17.14 [Source:ZFIN;Acc:ZDB-GENE-070912-153] | 3.16 | 4.21E-02 |
| ENSDART00000137862 | si:ch211-160b11.4 [Source:ZFIN;Acc:ZDB-GENE-081104-142] | 3.06 | 4.65E-02 |
| ENSDART00000020167 | solute carrier family 16 (monocarboxylic acid transporters), member 9a [Source:ZFIN;Acc:ZDB-GENE-040426-1364] | 2.86 | 1.11E-05 |
| ENSDART00000131126 | B-cell CLL/lymphoma 6a (zinc finger protein 51) [Source:ZFIN;Acc:ZDB-GENE-040426-1696] | 2.83 | 4.61E-03 |
| ENSDART00000114617 | Uncharacterized protein [Source:UniProtKB/TrEMBL;Acc:E9QGB0] | 2.82 | 1.99E-02 |
| ENSDART00000101326 | cytochrome P450, family 2, subfamily K, polypeptide 6 [Source:ZFIN;Acc:ZDB-GENE-040426-1571] | 2.68 | 5.39E-03 |
| ENSDART00000149039 | ornithine decarboxylase 1 [Source:ZFIN;Acc:ZDB-GENE-010816-1] | 2.65 | 1.83E-02 |
| ENSDART00000140230 | zgc:123068 [Source:ZFIN;Acc:ZDB-GENE-051030-98] | 2.59 | 1.85E-03 |
| ENSDART00000066142 | zgc:65811 [Source:ZFIN;Acc:ZDB-GENE-040426-1428] | 2.54 | 1.20E-04 |
| ENSDART00000056494 | microfibrillar-associated protein 4 [Source:HGNC Symbol;Acc:7035] | 2.52 | 2.39E-02 |
| ENSDART00000136903 | si:dkey-14d8.13 [Source:ZFIN;Acc:ZDB-GENE-041210-136] | 2.49 | 1.11E-03 |
| ENSDART00000077616 | solute carrier family 12, member 10.1 [Source:RefSeq peptide;Acc:NP_001154850] | 2.48 | 3.26E-02 |
| ENSDART00000133876 | si:dkey-250k15.9 [Source:ZFIN;Acc:ZDB-GENE-100922-41] | 2.43 | 3.78E-02 |
| ENSDART00000137962 | si:dkey-7f3.14 [Source:ZFIN;Acc:ZDB-GENE-100922-186] | 2.41 | 3.23E-03 |
| ENSDART00000033902 | PHD finger protein 11 [Source:HGNC Symbol;Acc:17024] | 2.37 | 1.57E-02 |
| ENSDART00000131544 | si:ch73-190m4.1 [Source:ZFIN;Acc:ZDB-GENE-090311-56] | 2.36 | 2.86E-02 |
| ENSDART00000130650 | hypothetical protein LOC100003647 [Source:RefSeq peptide;Acc:NP_001165779] | 2.33 | 2.60E-03 |
| ENSDART00000144582 | si:dkey-79f11.7 [Source:ZFIN;Acc:ZDB-GENE-090313-338] | 2.30 | 1.83E-03 |
| ENSDART00000149363 | serine/threonine kinase 25a [Source:ZFIN;Acc:ZDB-GENE-041010-92] | 2.29 | 5.42E-03 |
| ENSDART00000063476 | SRY-box containing gene 4b [Source:ZFIN;Acc:ZDB-GENE-040426-1274] | 2.26 | 2.49E-02 |
| ENSDART00000065512 | Uncharacterized protein [Source:UniProtKB/TrEMBL;Acc:E7F0D4] | 2.25 | 3.54E-02 |
| ENSDART00000146483 | si:dkey-188i13.9 [Source:ZFIN;Acc:ZDB-GENE-090313-211] | 2.23 | 1.59E-03 |
| ENSDART00000128863 | zgc:174356 [Source:ZFIN;Acc:ZDB-GENE-080215-21] | 2.22 | 5.36E-03 |
| ENSDART00000138176 | forkhead box D2 [Source:ZFIN;Acc:ZDB-GENE-980605-6] | 2.22 | 2.39E-02 |
| ENSDART00000138900 | si:ch211-207m11.3 [Source:ZFIN;Acc:ZDB-GENE-060503-210] | 2.21 | 2.74E-02 |
| ENSDART00000135515 | si:dkey-151g22.1 [Source:ZFIN;Acc:ZDB-GENE-041001-115] | 2.21 | 2.80E-02 |
| ENSDART00000139948 | chromosome 19 open reading frame 20 [Source:HGNC Symbol;Acc:25058] | 2.18 | 3.00E-02 |
| ENSDART00000116506 | microtubule-associated protein 7 [Source:HGNC Symbol;Acc:6869] | 2.12 | 1.82E-02 |
| ENSDART00000099591 | si:dkey-24c2.6 [Source:ZFIN;Acc:ZDB-GENE-070912-473] | 2.10 | 3.19E-02 |
| ENSDART00000083532 | solute carrier family 25, member 38a [Source:ZFIN;Acc:ZDB-GENE-060929-320] | 2.06 | 7.21E-03 |
| ENSDART00000111377 | solute carrier family 2 (facilitated glucose transporter), member 13 [Source:HGNC Symbol;Acc:15956] | 2.06 | 2.44E-02 |
| ENSDART00000112232 | TBC1 domain family, member 13 [Source:HGNC Symbol;Acc:25571] | 2.05 | 2.35E-02 |
| ENSDART00000060706 | RAD51 homolog (RecA homolog, E. coli) (S. cerevisiae) [Source:ZFIN;Acc:ZDB-GENE-040426-2286] | 2.05 | 3.95E-02 |
| ENSDART00000108855 | si:dkey-188i13.7 [Source:ZFIN;Acc:ZDB-GENE-090312-190] | 2.03 | 3.12E-03 |
| ENSDART00000017305 | zinc finger protein 76 [Source:HGNC Symbol;Acc:13149] | 2.02 | 2.31E-02 |
| ENSDART00000059918 | heterogeneous nuclear ribonucleoprotein H1 [Source:ZFIN;Acc:ZDB-GENE-040426-1856] | 2.02 | 3.78E-02 |
| ENSDART00000109495 | nudix (nucleoside diphosphate linked moiety X)-type motif 19 [Source:HGNC Symbol;Acc:32036] | 1.99 | 4.89E-02 |
| ENSDART00000141295 | si:dkey-188i13.11 [Source:ZFIN;Acc:ZDB-GENE-090313-209] | 1.98 | 1.00E-02 |
| ENSDART00000126215 | death associated protein 1b [Source:ZFIN;Acc:ZDB-GENE-000511-4] | 1.98 | 1.02E-02 |
| ENSDART00000145110 | lactase [Source:HGNC Symbol;Acc:6530] | 1.98 | 2.72E-03 |
| ENSDART00000130726 | BUB3 budding uninhibited by benzimidazoles 3 homolog (yeast) [Source:ZFIN;Acc:ZDB-GENE-041010-210] | 1.97 | 2.67E-02 |
| ENSDART00000144948 | si:dkey-33c9.4 [Source:ZFIN;Acc:ZDB-GENE-070727-1] | 1.95 | 2.12E-02 |
| ENSDART00000110792 | Uncharacterized protein [Source:UniProtKB/TrEMBL;Acc:E7F5P7] | 1.95 | 2.21E-02 |
| ENSDART00000100479 | solute carrier family 7 (cationic amino acid transporter, y+ system), member 9 [Source:ZFIN;Acc:ZDB-GENE-080116-2] | 1.95 | 2.13E-02 |
| ENSDART00000138285 | si:dkey-238c7.13 [Source:ZFIN;Acc:ZDB-GENE-030131-7311] | 1.93 | 2.92E-03 |
| ENSDART00000105040 | zgc:109868 [Source:ZFIN;Acc:ZDB-GENE-050417-363] | 1.91 | 1.32E-03 |
| ENSDART00000052076 | solute carrier family 30 (zinc transporter), member 10 [Source:ZFIN;Acc:ZDB-GENE-060608-2] | 1.91 | 6.96E-03 |
| ENSDART00000039780 | solute carrier family 1 (neutral amino acid transporter), member 5 [Source:ZFIN;Acc:ZDB-GENE-070501-4] | 1.91 | 4.29E-02 |
| ENSDART00000076405 | ghrelin/obestatin preprohormone [Source:ZFIN;Acc:ZDB-GENE-070622-2] | 1.90 | 3.67E-02 |
| ENSDART00000039055 | ring finger protein 170 [Source:ZFIN;Acc:ZDB-GENE-040426-2572] | 1.90 | 3.19E-03 |
| ENSDART00000124705 | zgc:110380 [Source:ZFIN;Acc:ZDB-GENE-050417-145] | 1.89 | 6.44E-03 |
| ENSDART00000144003 | solute carrier family 23 (nucleobase transporters), member 3 [Source:HGNC Symbol;Acc:20601] | 1.89 | 3.84E-02 |
| ENSDART00000082132 | F-box protein 7 [Source:ZFIN;Acc:ZDB-GENE-050913-68] | 1.88 | 1.56E-02 |
| ENSDART00000122520 | coiled-coil-helix-coiled-coil-helix domain containing 3 [Source:ZFIN;Acc:ZDB-GENE-030131-5005] | 1.88 | 2.17E-02 |
| ENSDART00000060266 | carcinoembryonic antigen-related cell adhesion molecule 1 [Source:ZFIN;Acc:ZDB-GENE-010724-14] | 1.87 | 1.21E-02 |
| ENSDART00000058171 | guanylate cyclase 2C (heat stable enterotoxin receptor) [Source:HGNC Symbol;Acc:4688] | 1.86 | 4.94E-02 |
| ENSDART00000132152 | si:ch211-69i14.10 [Source:ZFIN;Acc:ZDB-GENE-070705-187] | 1.85 | 1.76E-02 |
| ENSDART00000145554 | ribosomal protein L37 [Source:ZFIN;Acc:ZDB-GENE-040625-39] | 1.85 | 1.72E-03 |
| ENSDART00000052104 | fucosidase, alpha-L- 1, tissue [Source:ZFIN;Acc:ZDB-GENE-030131-7434] | 1.84 | 3.09E-03 |
| ENSDART00000041806 | acyl-CoA synthetase medium-chain family member 3 [Source:ZFIN;Acc:ZDB-GENE-080220-22] | 1.83 | 2.98E-02 |
| ENSDART00000023162 | Fanconi anemia, complementation group A [Source:HGNC Symbol;Acc:3582] | 1.83 | 1.75E-02 |
| ENSDART00000053892 | chromosome 7 open reading frame 55 [Source:HGNC Symbol;Acc:26946] | 1.82 | 2.61E-02 |
| ENSDART00000063175 | Uncharacterized protein [Source:UniProtKB/TrEMBL;Acc:F1RC17] | 1.82 | 3.19E-02 |
| ENSDART00000063912 | jun proto-oncogene [Source:ZFIN;Acc:ZDB-GENE-030131-7859] | 1.81 | 3.26E-02 |
| ENSDART00000063870 | ribosomal protein L11 [Source:ZFIN;Acc:ZDB-GENE-040625-147] | 1.80 | 2.99E-03 |
| ENSDART00000006942 | thioredoxin domain containing 15 [Source:HGNC Symbol;Acc:20652] | 1.79 | 1.52E-02 |
| ENSDART00000054594 | cytokine receptor family member b5 [Source:ZFIN;Acc:ZDB-GENE-050909-1] | 1.79 | 9.15E-03 |
| ENSDART00000115257 | Uncharacterized protein [Source:UniProtKB/TrEMBL;Acc:E7FBQ2] | 1.79 | 4.31E-02 |
| ENSDART00000115136 | DENN/MADD domain containing 3a [Source:ZFIN;Acc:ZDB-GENE-091230-6] | 1.78 | 2.97E-02 |
| ENSDART00000002812 | castor zinc finger 1 [Source:ZFIN;Acc:ZDB-GENE-060130-108] | 1.77 | 3.85E-02 |
| ENSDART00000057945 | ATPase, H+ transporting, lysosomal 9kDa, V0 subunit e1 [Source:HGNC Symbol;Acc:863] | 1.76 | 2.28E-02 |
| ENSDART00000004780 | mannosidase, alpha, class 2B, member 1 [Source:HGNC Symbol;Acc:6826] | 1.76 | 3.16E-02 |
| ENSDART00000077910 | si:dkey-270m17.5 [Source:ZFIN;Acc:ZDB-GENE-091204-250] | 1.76 | 8.04E-03 |
| ENSDART00000005903 | ATP synthase, H+ transporting, mitochondrial F0 complex, subunit c (subunit 9) [Source:ZFIN;Acc:ZDB-GENE-020814-1] | 1.75 | 5.75E-03 |
| ENSDART00000021406 | peptidylprolyl isomerase (cyclophilin)-like 1 [Source:ZFIN;Acc:ZDB-GENE-051009-1] | 1.75 | 1.71E-02 |
| ENSDART00000077436 | myosin regulatory light chain interacting protein b [Source:ZFIN;Acc:ZDB-GENE-061027-67] | 1.75 | 2.31E-02 |
| ENSDART00000067558 | spastic paraplegia 11 [Source:ZFIN;Acc:ZDB-GENE-101017-1] | 1.74 | 2.53E-02 |
| ENSDART00000097263 | capthepsin B, b [Source:ZFIN;Acc:ZDB-GENE-070323-1] | 1.74 | 4.06E-02 |
| ENSDART00000147520 | ligand of numb-protein X 1 [Source:ZFIN;Acc:ZDB-GENE-030131-9439] | 1.73 | 4.06E-02 |
| ENSDART00000063359 | uncoupling protein 2 [Source:ZFIN;Acc:ZDB-GENE-990708-8] | 1.73 | 1.14E-02 |
| ENSDART00000100757 | oxidase (cytochrome c) assembly 1-like [Source:ZFIN;Acc:ZDB-GENE-071004-49] | 1.73 | 4.81E-02 |
| ENSDART00000076731 | acyl-CoA thioesterase 12 [Source:HGNC Symbol;Acc:24436] | 1.72 | 3.41E-02 |
| ENSDART00000037904 | suppressor of cytokine signaling 3b [Source:ZFIN;Acc:ZDB-GENE-040426-2528] | 1.72 | 3.70E-02 |
| ENSDART00000105878 | filamin binding LIM protein 1 [Source:HGNC Symbol;Acc:24686] | 1.71 | 1.19E-02 |
| ENSDART00000080832 | si:dkey-6n6.2 [Source:ZFIN;Acc:ZDB-GENE-060503-173] | 1.71 | 2.63E-02 |
| ENSDART00000067866 | ribosomal protein S17 [Source:HGNC Symbol;Acc:10397] | 1.71 | 8.12E-03 |
| ENSDART00000073462 | ribosomal protein, large, P0 [Source:ZFIN;Acc:ZDB-GENE-000629-1] | 1.70 | 6.09E-03 |
| ENSDART00000112803 | Uncharacterized protein [Source:UniProtKB/TrEMBL;Acc:E7FA76] | 1.70 | 4.98E-02 |
| ENSDART00000139430 | complement component 6 [Source:ZFIN;Acc:ZDB-GENE-040426-1358] | 1.69 | 3.01E-02 |
| ENSDART00000141100 | ribosomal protein S2 [Source:ZFIN;Acc:ZDB-GENE-040426-2454] | 1.69 | 6.38E-03 |
| ENSDART00000132168 | zinc finger protein 410 [Source:ZFIN;Acc:ZDB-GENE-040426-1817] | 1.69 | 2.94E-02 |
| ENSDART00000008215 | phosphatidylinositol-4-phosphate 5-kinase, type I, beta [Source:HGNC Symbol;Acc:8995] | 1.69 | 2.65E-02 |
| ENSDART00000100170 | glutamate-ammonia ligase (glutamine synthase) a [Source:ZFIN;Acc:ZDB-GENE-030131-688] | 1.69 | 2.34E-02 |
| ENSDART00000024407 | transmembrane and immunoglobulin domain containing 1 [Source:HGNC Symbol;Acc:32431] | 1.69 | 1.61E-02 |
| ENSDART00000063285 | latexin [Source:ZFIN;Acc:ZDB-GENE-041212-18] | 1.68 | 1.41E-02 |
| ENSDART00000046840 | exosome component 3 [Source:ZFIN;Acc:ZDB-GENE-050706-140] | 1.67 | 4.07E-02 |
| ENSDART00000102855 | pyruvate dehydrogenase complex, component X [Source:ZFIN;Acc:ZDB-GENE-040426-1539] | 1.67 | 3.36E-02 |
| ENSDART00000048599 | ribosomal protein S19 [Source:ZFIN;Acc:ZDB-GENE-040426-1716] | 1.67 | 8.40E-03 |
| ENSDART00000060990 | eukaryotic translation initiation factor 4E binding protein 3, like [Source:ZFIN;Acc:ZDB-GENE-030826-26] | 1.67 | 1.76E-02 |
| ENSDART00000055395 | odd-skipped related 2 (Drosophila) [Source:ZFIN;Acc:ZDB-GENE-050417-183] | 1.67 | 4.77E-02 |
| ENSDART00000054469 | S100 calcium binding protein A10a [Source:ZFIN;Acc:ZDB-GENE-041010-35] | 1.66 | 1.23E-02 |
| ENSDART00000057423 | telomeric repeat binding factor a [Source:ZFIN;Acc:ZDB-GENE-020419-38] | 1.66 | 3.69E-02 |
| ENSDART00000125936 | N-acetylgalactosaminidase, alpha- [Source:HGNC Symbol;Acc:7631] | 1.66 | 1.33E-02 |
| ENSDART00000025229 | acireductone dioxygenase 1 [Source:ZFIN;Acc:ZDB-GENE-030131-2799] | 1.65 | 3.89E-02 |
| ENSDART00000114203 | mannosidase, alpha, class 2B, member 2 [Source:HGNC Symbol;Acc:29623] | 1.65 | 3.00E-02 |
| ENSDART00000136655 | NADH dehydrogenase (ubiquinone) 1 alpha subcomplex, 3, 9kDa [Source:HGNC Symbol;Acc:7686] | 1.65 | 2.55E-02 |
| ENSDART00000141397 | ribosomal protein L15 [Source:ZFIN;Acc:ZDB-GENE-040801-183] | 1.64 | 1.07E-02 |
| ENSDART00000045901 | ribosomal protein S10 [Source:ZFIN;Acc:ZDB-GENE-040426-1481] | 1.64 | 1.20E-02 |
| ENSDART00000125391 | solute carrier family 25, member 34 [Source:HGNC Symbol;Acc:27653] | 1.64 | 3.11E-02 |
| ENSDART00000112103 | glucosamine (N-acetyl)-6-sulfatase (Sanfilippo disease IIID), b [Source:ZFIN;Acc:ZDB-GENE-030131-5846] | 1.63 | 2.54E-02 |
| ENSDART00000082325 | angio-associated, migratory cell protein [Source:HGNC Symbol;Acc:18] | 1.63 | 2.60E-02 |
| ENSDART00000145781 | si:ch211-160e1.5 [Source:ZFIN;Acc:ZDB-GENE-070705-255] | 1.63 | 4.79E-02 |
| ENSDART00000134516 | basic transcription factor 3 [Source:ZFIN;Acc:ZDB-GENE-030131-8731] | 1.63 | 1.16E-02 |
| ENSDART00000121770 | ribosomal protein L35a [Source:ZFIN;Acc:ZDB-GENE-040718-190] | 1.63 | 1.29E-02 |
| ENSDART00000081505 | protein geranylgeranyltransferase type I, beta subunit [Source:ZFIN;Acc:ZDB-GENE-050913-85] | 1.63 | 2.89E-02 |
| ENSDART00000142945 | pallidin homolog (mouse) [Source:ZFIN;Acc:ZDB-GENE-070112-1862] | 1.63 | 1.90E-02 |
| ENSDART00000052082 | ribosomal protein L30 [Source:ZFIN;Acc:ZDB-GENE-030131-8657] | 1.62 | 1.29E-02 |
| ENSDART00000041279 | tubulin, beta 2c [Source:ZFIN;Acc:ZDB-GENE-030131-8625] | 1.61 | 1.87E-02 |
| ENSDART00000133990 | solute carrier family 25 alpha, member 5 [Source:ZFIN;Acc:ZDB-GENE-020419-9] | 1.61 | 3.16E-02 |
| ENSDART00000047531 | chloride channel accessory 2 [Source:HGNC Symbol;Acc:2016] | 1.61 | 3.97E-02 |
| ENSDART00000148052 | si:ch211-245h7.2 [Source:ZFIN;Acc:ZDB-GENE-030131-1521] | 1.61 | 4.81E-02 |
| ENSDART00000038099 | KLRAQ motif containing 1 [Source:HGNC Symbol;Acc:30595] | 1.61 | 3.47E-02 |
| ENSDART00000043076 | pancreatic progenitor cell differentiation and proliferation factor b [Source:ZFIN;Acc:ZDB-GENE-030131-8247] | 1.61 | 3.55E-02 |
| ENSDART00000033237 | wdr45 like [Source:ZFIN;Acc:ZDB-GENE-040426-863] | 1.60 | 2.56E-02 |
| ENSDART00000014473 | cytochrome c oxidase subunit Va [Source:HGNC Symbol;Acc:2267] | 1.60 | 3.73E-02 |
| ENSDART00000090644 | mitochondrial ribosomal protein L2 [Source:HGNC Symbol;Acc:14056] | 1.60 | 2.99E-02 |
| ENSDART00000087242 | ATPase inhibitory factor 1 [Source:ZFIN;Acc:ZDB-GENE-070410-36] | 1.59 | 4.98E-02 |
| ENSDART00000077386 | protease, serine, 16 (thymus) [Source:HGNC Symbol;Acc:9480] | 1.59 | 3.13E-02 |
| ENSDART00000056286 | H1 histone family, member 0 [Source:HGNC Symbol;Acc:4714] | 1.59 | 4.49E-02 |
| ENSDART00000062153 | ribosomal protein L37a [Source:HGNC Symbol;Acc:10348] | 1.58 | 1.89E-02 |
| ENSDART00000138350 | ribosomal protein S12 [Source:ZFIN;Acc:ZDB-GENE-030131-8951] | 1.58 | 1.83E-02 |
| ENSDART00000104364 | ribosomal protein S15 [Source:ZFIN;Acc:ZDB-GENE-030131-9092] | 1.57 | 2.07E-02 |
| ENSDART00000110456 | non-metastatic cells 2b.1, protein (NM23B) expressed in [Source:ZFIN;Acc:ZDB-GENE-000210-32] | 1.57 | 2.31E-02 |
| ENSDART00000100281 | aldehyde dehydrogenase 9 family, member A1 [Source:HGNC Symbol;Acc:412] | 1.56 | 4.83E-02 |
| ENSDART00000075958 | solute carrier family 13 (sodium-dependent dicarboxylate transporter), member 2  [Source:ZFIN;Acc:ZDB-GENE-040426-2389] | 1.56 | 2.35E-02 |
| ENSDART00000010658 | serpin peptidase inhibitor, clade B (ovalbumin), member 1, like 3 [Source:ZFIN;Acc:ZDB-GENE-030131-7059] | 1.56 | 2.97E-02 |
| ENSDART00000015277 | glutathione peroxidase 1b [Source:ZFIN;Acc:ZDB-GENE-040912-60] | 1.56 | 2.63E-02 |
| ENSDART00000067803 | ribosomal protein S3 [Source:ZFIN;Acc:ZDB-GENE-030131-8494] | 1.55 | 2.26E-02 |
| ENSDART00000128025 | ribosomal protein S26, like [Source:ZFIN;Acc:ZDB-GENE-040426-1706] | 1.54 | 2.73E-02 |
| ENSDART00000127198 | ribosomal protein S26 [Source:ZFIN;Acc:ZDB-GENE-030131-8606] | 1.54 | 2.82E-02 |
| ENSDART00000011435 | cytochrome c oxidase subunit Vab [Source:ZFIN;Acc:ZDB-GENE-030131-5162] | 1.54 | 4.30E-02 |
| ENSDART00000063800 | glyceraldehyde-3-phosphate dehydrogenase [Source:ZFIN;Acc:ZDB-GENE-030115-1] | 1.53 | 2.69E-02 |
| ENSDART00000140583 | ribosomal protein L17 [Source:HGNC Symbol;Acc:10307] | 1.53 | 2.83E-02 |
| ENSDART00000001201 | bystin-like [Source:ZFIN;Acc:ZDB-GENE-040426-1287] | 1.53 | 4.90E-02 |
| ENSDART00000008243 | slowmo homolog 2 (Drosophila) [Source:ZFIN;Acc:ZDB-GENE-031002-13] | 1.52 | 4.38E-02 |
| ENSDART00000102251 | spectrin alpha 2 [Source:ZFIN;Acc:ZDB-GENE-051113-60] | 1.52 | 4.95E-02 |
| ENSDART00000064700 | fucosidase, alpha-L- 2, plasma [Source:ZFIN;Acc:ZDB-GENE-040822-39] | 1.52 | 4.07E-02 |
| ENSDART00000010395 | ubiquinol-cytochrome c reductase core protein IIa [Source:ZFIN;Acc:ZDB-GENE-040718-405] | 1.52 | 4.49E-02 |
| ENSDART00000075028 | ribosomal protein S11 [Source:ZFIN;Acc:ZDB-GENE-040426-2701] | 1.51 | 3.27E-02 |
| ENSDART00000150204 | ATP synthase, H+ transporting, mitochondrial F1 complex, epsilon subunit pseudogene 2 [Source:HGNC Symbol;Acc:34026] | 1.51 | 4.39E-02 |
| ENSDART00000051763 | ribosomal protein S3A [Source:ZFIN;Acc:ZDB-GENE-030131-9184] | 1.51 | 3.43E-02 |
| ENSDART00000077834 | zgc:73262 [Source:ZFIN;Acc:ZDB-GENE-040426-1735] | 1.50 | 4.31E-02 |
| ENSDART00000005944 | ribosomal protein L5a [Source:ZFIN;Acc:ZDB-GENE-030131-5161] | 1.50 | 3.72E-02 |
| ENSDART00000013690 | ribosomal protein, large P2, like [Source:ZFIN;Acc:ZDB-GENE-070327-2] | 1.50 | 4.37E-02 |
| ENSDART00000110777 | elongation factor-1, delta, b [Source:ZFIN;Acc:ZDB-GENE-030131-6544] | 1.49 | 4.66E-02 |
| ENSDART00000115195 | eukaryotic translation initiation factor 3, subunit F [Source:HGNC Symbol;Acc:3275] | 1.49 | 4.00E-02 |
| ENSDART00000075495 | ribosomal protein L23 [Source:ZFIN;Acc:ZDB-GENE-030131-8756] | 1.49 | 4.30E-02 |
| ENSDART00000040204 | tubulin, alpha 8 like 2 [Source:ZFIN;Acc:ZDB-GENE-040426-1646] | 1.49 | 4.03E-02 |
| ENSDART00000054338 | ribosomal protein L9 [Source:ZFIN;Acc:ZDB-GENE-030131-8646] | 1.49 | 3.98E-02 |
| ENSDART00000060745 | ubiquitin A-52 residue ribosomal protein fusion product 1 [Source:ZFIN;Acc:ZDB-GENE-051023-7] | 1.49 | 4.34E-02 |
| ENSDART00000031173 | zgc:109888 [Source:ZFIN;Acc:ZDB-GENE-050522-549] | 1.49 | 4.15E-02 |
| ENSDART00000025620 | peptidylprolyl isomerase A (cyclophilin A) [Source:ZFIN;Acc:ZDB-GENE-030131-8556] | 1.49 | 3.96E-02 |
| ENSDART00000061001 | guanine nucleotide binding protein (G protein), beta polypeptide 2-like 1 [Source:ZFIN;Acc:ZDB-GENE-990415-89] | 1.48 | 4.30E-02 |
| ENSDART00000140039 | ribosomal protein L8 [Source:ZFIN;Acc:ZDB-GENE-040426-1670] | 1.48 | 4.10E-02 |
| ENSDART00000004692 | isocitrate dehydrogenase 2 (NADP+), mitochondrial [Source:HGNC Symbol;Acc:5383] | 1.48 | 4.94E-02 |
| ENSDART00000063019 | muscle-specific beta 1 integrin binding protein 2 [Source:ZFIN;Acc:ZDB-GENE-031113-14] | 1.48 | 4.43E-02 |
| ENSDART00000019227 | ribosomal protein L24 [Source:ZFIN;Acc:ZDB-GENE-020419-25] | 1.48 | 4.98E-02 |
| ENSDART00000140784 | si:dkey-238c7.12 [Source:ZFIN;Acc:ZDB-GENE-030131-8330] | 1.48 | 4.45E-02 |
| ENSDART00000030215 | ribosomal protein L18a [Source:ZFIN;Acc:ZDB-GENE-040426-1071] | 1.47 | 4.36E-02 |
| ENSDART00000015199 | ribosomal protein L36 [Source:ZFIN;Acc:ZDB-GENE-040622-2] | 1.47 | 4.54E-02 |
| ENSDART00000030482 | sulfotransferase family 1, cytosolic sulfotransferase 2 [Source:ZFIN;Acc:ZDB-GENE-030804-27] | 0.66 | 4.27E-02 |
| ENSDART00000141714 | nuclear protein 1 [Source:ZFIN;Acc:ZDB-GENE-030131-4653] | 0.64 | 3.71E-02 |
| ENSDART00000145427 | synaptophysin-like 2a [Source:ZFIN;Acc:ZDB-GENE-040426-1434] | 0.63 | 4.27E-02 |
| ENSDART00000133308 | nidogen 1a [Source:ZFIN;Acc:ZDB-GENE-050302-58] | 0.62 | 4.03E-02 |
| ENSDART00000004664 | translocating chain-associating membrane protein 1 [Source:ZFIN;Acc:ZDB-GENE-021011-1] | 0.62 | 2.71E-02 |
| ENSDART00000112204 | trafficking protein, kinesin binding 2 [Source:HGNC Symbol;Acc:13206] | 0.61 | 3.88E-02 |
| ENSDART00000103755 | fibronectin 1b [Source:ZFIN;Acc:ZDB-GENE-030131-6545] | 0.61 | 2.90E-02 |
| ENSDART00000136402 | zgc:56585 [Source:ZFIN;Acc:ZDB-GENE-040426-1084] | 0.60 | 1.57E-02 |
| ENSDART00000037287 | Uncharacterized protein [Source:UniProtKB/TrEMBL;Acc:E7F727] | 0.60 | 3.67E-02 |
| ENSDART00000140752 | myo-inositol oxygenase [Source:ZFIN;Acc:ZDB-GENE-050913-113] | 0.60 | 2.64E-02 |
| ENSDART00000123374 | DnaJ (Hsp40) homolog, subfamily B, member 11 [Source:ZFIN;Acc:ZDB-GENE-031113-9] | 0.59 | 2.24E-02 |
| ENSDART00000133447 | si:ch211-288g17.4 [Source:ZFIN;Acc:ZDB-GENE-060531-62] | 0.59 | 4.88E-02 |
| ENSDART00000020942 | sex hormone binding globulin [Source:ZFIN;Acc:ZDB-GENE-030131-1324] | 0.59 | 2.60E-02 |
| ENSDART00000141249 | si:dkey-236e20.2 [Source:ZFIN;Acc:ZDB-GENE-060503-628] | 0.58 | 3.55E-02 |
| ENSDART00000138595 | complement component 3 [Source:HGNC Symbol;Acc:1318] | 0.58 | 3.45E-02 |
| ENSDART00000114795 | dihydropyrimidinase [Source:ZFIN;Acc:ZDB-GENE-070508-1] | 0.57 | 3.41E-02 |
| ENSDART00000060049 | heat shock protein 13 [Source:ZFIN;Acc:ZDB-GENE-070410-58] | 0.56 | 3.76E-02 |
| ENSDART00000143723 | isopentenyl-diphosphate delta isomerase 1 [Source:ZFIN;Acc:ZDB-GENE-050913-44] | 0.56 | 3.94E-02 |
| ENSDART00000010449 | UDP-glucose pyrophosphorylase 2a [Source:ZFIN;Acc:ZDB-GENE-090311-46] | 0.56 | 4.81E-02 |
| ENSDART00000148929 | si:ch73-226l13.2 [Source:ZFIN;Acc:ZDB-GENE-110411-18] | 0.55 | 9.80E-03 |
| ENSDART00000078734 | alpha-2-macroglobulin-like [Source:ZFIN;Acc:ZDB-GENE-090212-1] | 0.55 | 3.38E-02 |
| ENSDART00000025570 | calreticulin like [Source:ZFIN;Acc:ZDB-GENE-030131-9907] | 0.55 | 5.87E-03 |
| ENSDART00000074327 | insulin-like growth factor binding protein 2a [Source:ZFIN;Acc:ZDB-GENE-000125-12] | 0.55 | 2.35E-02 |
| ENSDART00000134931 | 3-hydroxy-3-methylglutaryl-Coenzyme A synthase 1 (soluble) [Source:ZFIN;Acc:ZDB-GENE-040426-1042] | 0.55 | 2.88E-02 |
| ENSDART00000077296 | collagen, type IV, alpha 1 [Source:ZFIN;Acc:ZDB-GENE-081105-114] | 0.55 | 4.48E-02 |
| ENSDART00000132121 | zgc:110377 [Source:ZFIN;Acc:ZDB-GENE-050522-262] | 0.54 | 1.10E-02 |
| ENSDART00000130970 | syncollin [Source:HGNC Symbol;Acc:18442] | 0.54 | 2.26E-02 |
| ENSDART00000093611 | ATP synthase 8, mitochondrial [Source:ZFIN;Acc:ZDB-GENE-011205-19] | 0.54 | 2.80E-03 |
| ENSDART00000103347 | chromosome 14 open reading frame 1 [Source:HGNC Symbol;Acc:1187] | 0.53 | 7.93E-03 |
| ENSDART00000149281 | solute carrier family 16 (monocarboxylic acid transporters), member 12a [Source:ZFIN;Acc:ZDB-GENE-080721-24] | 0.52 | 1.79E-02 |
| ENSDART00000146018 | methylenetetrahydrofolate reductase (NAD(P)H) [Source:HGNC Symbol;Acc:7436] | 0.51 | 1.25E-02 |
| ENSDART00000138719 | cytochrome P450, family 3, subfamily C, polypeptide 4 [Source:ZFIN;Acc:ZDB-GENE-060929-96] | 0.51 | 2.42E-02 |
| ENSDART00000113505 | myosin, light polypeptide 9, like [Source:ZFIN;Acc:ZDB-GENE-040426-2296] | 0.50 | 2.32E-02 |
| ENSDART00000133904 | si:ch211-274f20.2 [Source:ZFIN;Acc:ZDB-GENE-091204-91] | 0.50 | 2.77E-02 |
| ENSDART00000121764 | one cut domain, family member, like [Source:ZFIN;Acc:ZDB-GENE-020531-1] | 0.48 | 2.40E-02 |
| ENSDART00000121589 | starch binding domain 1 [Source:HGNC Symbol;Acc:24854] | 0.48 | 1.09E-02 |
| ENSDART00000147198 | insulin-like growth factor binding protein, acid labile subunit [Source:HGNC Symbol;Acc:5468] | 0.48 | 2.07E-02 |
| ENSDART00000053921 | solute carrier family 7 (cationic amino acid transporter, y+ system), member 2 [Source:ZFIN;Acc:ZDB-GENE-041212-5] | 0.46 | 3.24E-03 |
| ENSDART00000089332 | acyl-CoA synthetase bubblegum family member 1 [Source:HGNC Symbol;Acc:29567] | 0.46 | 3.46E-02 |
| ENSDART00000131742 | solute carrier family 5 (sodium/myo-inositol cotransporter), member 3 [Source:HGNC Symbol;Acc:11038] | 0.46 | 3.25E-02 |
| ENSDART00000132255 | si:ch73-15n24.1 [Source:ZFIN;Acc:ZDB-GENE-030131-1323] | 0.45 | 2.56E-02 |
| ENSDART00000083582 | sterol O-acyltransferase 2 [Source:HGNC Symbol;Acc:11178] | 0.44 | 1.88E-02 |
| ENSDART00000040383 | Uncharacterized protein [Source:UniProtKB/TrEMBL;Acc:E7FAM3] | 0.43 | 2.07E-02 |
| ENSDART00000045921 | furry homolog a (Drosophila) [Source:ZFIN;Acc:ZDB-GENE-060510-4] | 0.43 | 2.55E-02 |
| ENSDART00000060150 | MID1 interacting protein 1a [Source:ZFIN;Acc:ZDB-GENE-990415-81] | 0.43 | 3.03E-02 |
| ENSDART00000003242 | phospholipase A2, group III [Source:HGNC Symbol;Acc:17934] | 0.43 | 1.32E-02 |
| ENSDART00000141745 | si:dkey-150i13.2 [Source:ZFIN;Acc:ZDB-GENE-090312-157] | 0.43 | 2.97E-03 |
| ENSDART00000009393 | collagen, type I, alpha 1a [Source:ZFIN;Acc:ZDB-GENE-030131-9102] | 0.43 | 4.24E-03 |
| ENSDART00000126929 | Uncharacterized protein [Source:UniProtKB/TrEMBL;Acc:E7F608] | 0.43 | 2.15E-02 |
| ENSDART00000146779 | solute carrier family 38, member 2 [Source:ZFIN;Acc:ZDB-GENE-030131-9659] | 0.42 | 3.43E-04 |
| ENSDART00000129427 | vitamin K epoxide reductase complex, subunit 1 [Source:HGNC Symbol;Acc:23663] | 0.42 | 4.02E-02 |
| ENSDART00000013001 | zgc:152753 [Source:ZFIN;Acc:ZDB-GENE-060818-8] | 0.41 | 3.75E-02 |
| ENSDART00000113553 | apolipoprotein B (including Ag(x) antigen) [Source:HGNC Symbol;Acc:603] | 0.41 | 3.38E-04 |
| ENSDART00000063625 | glutathione peroxidase 3 (plasma) [Source:ZFIN;Acc:ZDB-GENE-070222-3] | 0.40 | 3.51E-03 |
| ENSDART00000099426 | cytokine receptor family member b1 [Source:ZFIN;Acc:ZDB-GENE-030131-8673] | 0.40 | 1.06E-02 |
| ENSDART00000077080 | protein tyrosine phosphatase type IVA, member 3 [Source:HGNC Symbol;Acc:9636] | 0.40 | 8.74E-03 |
| ENSDART00000127312 | oxidative stress induced growth inhibitor 1 [Source:HGNC Symbol;Acc:30093] | 0.38 | 1.18E-04 |
| ENSDART00000123104 | notch homolog 2 [Source:ZFIN;Acc:ZDB-GENE-000329-4] | 0.36 | 3.33E-04 |
| ENSDART00000063320 | claudin e [Source:ZFIN;Acc:ZDB-GENE-010328-5] | 0.36 | 2.20E-02 |
| ENSDART00000076850 | claudin i [Source:ZFIN;Acc:ZDB-GENE-010328-9] | 0.35 | 2.73E-02 |
| ENSDART00000085763 | zgc:165571 [Source:ZFIN;Acc:ZDB-GENE-030131-7249] | 0.35 | 4.52E-02 |
| ENSDART00000135359 | metastasis associated in colon cancer 1 [Source:ZFIN;Acc:ZDB-GENE-091006-3] | 0.35 | 2.00E-02 |
| ENSDART00000078894 | interferon regulatory factor 8 [Source:ZFIN;Acc:ZDB-GENE-040718-367] | 0.35 | 3.37E-02 |
| ENSDART00000127194 | growth hormone receptor a [Source:ZFIN;Acc:ZDB-GENE-070509-1] | 0.34 | 3.64E-02 |
| ENSDART00000037017 | zgc:165543 [Source:ZFIN;Acc:ZDB-GENE-070615-25] | 0.34 | 7.79E-03 |
| ENSDART00000104800 | peptidoglycan recognition protein 6 [Source:ZFIN;Acc:ZDB-GENE-071227-2] | 0.33 | 9.49E-05 |
| ENSDART00000041728 | cytochrome P450, subfamily XXVIA, polypeptide 1 [Source:ZFIN;Acc:ZDB-GENE-990415-44] | 0.32 | 3.79E-02 |
| ENSDART00000126444 | matrix metalloproteinase 13a [Source:ZFIN;Acc:ZDB-GENE-031202-2] | 0.32 | 3.97E-02 |
| ENSDART00000124856 | Uncharacterized protein [Source:UniProtKB/TrEMBL;Acc:E7F2V9] | 0.30 | 6.54E-03 |
| ENSDART00000098045 | growth arrest-specific 1b [Source:ZFIN;Acc:ZDB-GENE-050302-79] | 0.29 | 3.82E-02 |
| ENSDART00000146717 | chloride intracellular channel a [Source:ZFIN;Acc:ZDB-GENE-010507-2] | 0.28 | 4.59E-02 |
| ENSDART00000129818 | phosphofurin acidic cluster sorting protein 1 [Source:HGNC Symbol;Acc:30032] | 0.28 | 4.30E-02 |
| ENSDART00000126123 | Uncharacterized protein [Source:UniProtKB/TrEMBL;Acc:E7FCS0] | 0.27 | 2.15E-02 |
| ENSDART00000125864 | calcium/calmodulin-dependent protein kinase kinase 1, alpha [Source:HGNC Symbol;Acc:1469] | 0.27 | 4.94E-02 |
| ENSDART00000022625 | notch-regulated ankyrin repeat protein b [Source:ZFIN;Acc:ZDB-GENE-030515-7] | 0.27 | 1.89E-02 |
| ENSDART00000135029 | si:dkey-270e21.7 [Source:ZFIN;Acc:ZDB-GENE-050208-798] | 0.26 | 3.37E-02 |
| ENSDART00000102554 | low density lipoprotein receptor-related protein 8, apolipoprotein e receptor [Source:HGNC Symbol;Acc:6700] | 0.25 | 3.15E-02 |
| ENSDART00000034549 | mucin 17, cell surface associated [Source:HGNC Symbol;Acc:16800] | 0.25 | 1.14E-05 |
| ENSDART00000135192 | zgc:100868 [Source:ZFIN;Acc:ZDB-GENE-040801-33] | 0.25 | 5.25E-03 |
| ENSDART00000053440 | Uncharacterized protein [Source:UniProtKB/TrEMBL;Acc:E7F126] | 0.23 | 1.80E-02 |
| ENSDART00000063760 | Uncharacterized protein [Source:UniProtKB/TrEMBL;Acc:E7F2A5] | 0.22 | 3.69E-04 |
| ENSDART00000126322 | Uncharacterized protein [Source:UniProtKB/TrEMBL;Acc:E7EZG1] | 0.21 | 4.89E-03 |
| ENSDART00000144497 | piwi-like 1 (Drosophila) [Source:ZFIN;Acc:ZDB-GENE-030813-2] | 0.20 | 3.21E-02 |
| ENSDART00000134959 | si:dkey-30j22.1 [Source:ZFIN;Acc:ZDB-GENE-041001-184] | 0.20 | 1.73E-02 |
| ENSDART00000102316 | elastin a [Source:ZFIN;Acc:ZDB-GENE-061212-1] | 0.19 | 4.92E-03 |
| ENSDART00000148436 | guanine nucleotide binding protein (G protein), alpha 14 [Source:ZFIN;Acc:ZDB-GENE-040808-7] | 0.19 | 3.55E-02 |
| ENSDART00000114023 | heat shock protein, alpha-crystallin-related, 9 [Source:ZFIN;Acc:ZDB-GENE-080214-6] | 0.19 | 4.78E-02 |
| ENSDART00000027839 | solute carrier family 12, member 1 [Source:ZFIN;Acc:ZDB-GENE-080130-3] | 0.18 | 1.01E-02 |
| ENSDART00000149095 | vasa homolog [Source:ZFIN;Acc:ZDB-GENE-990415-272] | 0.18 | 2.16E-02 |
| ENSDART00000019846 | collagen, type VIII, alpha 1b [Source:ZFIN;Acc:ZDB-GENE-060503-140] | 0.15 | 2.86E-02 |
| ENSDART00000139802 | si:ch211-173p18.2 [Source:ZFIN;Acc:ZDB-GENE-060503-578] | 0.14 | 3.40E-02 |
| ENSDART00000011970 | zgc:66479 [Source:ZFIN;Acc:ZDB-GENE-031030-10] | 0.14 | 3.66E-02 |
| ENSDART00000128412 | Uncharacterized protein [Source:UniProtKB/TrEMBL;Acc:E7FAC1] | 0.13 | 4.59E-02 |
| ENSDART00000053382 | upregulator of cell proliferation [Source:HGNC Symbol;Acc:30890] | 0.09 | 2.01E-02 |
| ENSDART00000136505 | guanine nucleotide binding protein (G protein), alpha inhibiting activity polypeptide 3  [Source:ZFIN;Acc:ZDB-GENE-070713-6] | 0.07 | 4.92E-02 |
| ENSDART00000139366 | si:ch211-238e22.1 [Source:ZFIN;Acc:ZDB-GENE-041210-345] | 0.05 | 7.25E-04 |
| ENSDART00000111190 | si:dkey-238f9.1 [Source:ZFIN;Acc:ZDB-GENE-091204-297] | 0.04 | 4.97E-02 |
| ENSDART00000102298 | scinderin like a [Source:ZFIN;Acc:ZDB-GENE-030131-2005] | 0.04 | 4.64E-02 |
| ENSDART00000104256 | optic atrophy 1 (autosomal dominant) [Source:HGNC Symbol;Acc:8140] | 0.04 | 6.05E-06 |
| ENSDART00000146919 | si:dkeyp-82a1.1 [Source:ZFIN;Acc:ZDB-GENE-081105-23] | 0.04 | 8.36E-03 |
| ENSDART00000142365 | si:ch211-278f9.2 [Source:ZFIN;Acc:ZDB-GENE-090311-4] | 0.04 | 4.07E-02 |
| ENSDART00000113109 | leucine-rich repeat LGI family, member 2b [Source:ZFIN;Acc:ZDB-GENE-060217-3] | 0.03 | 4.03E-02 |
| ENSDART00000131404 | si:ch211-159e21.1 [Source:ZFIN;Acc:ZDB-GENE-091116-99] | 0.01 | 5.36E-03 |
| ENSDART00000141876 | finTRIM family, member 30 [Source:ZFIN;Acc:ZDB-GENE-070912-106] | 0.01 | 2.18E-02 |
| ENSDART00000114886 | secretogranin V (7B2 protein) [Source:HGNC Symbol;Acc:10816] | 0.00 | 1.24E-07 |
| ENSDART00000109325 | Uncharacterized protein [Source:UniProtKB/TrEMBL;Acc:F1RDV1] | 0.00 | 7.73E-03 |
| ENSDART00000141635 | si:ch211-229n2.6 [Source:ZFIN;Acc:ZDB-GENE-081105-166] | 0.00 | 8.84E-03 |
| ENSDART00000075859 | zgc:172065 [Source:ZFIN;Acc:ZDB-GENE-080214-2] | 0.00 | 1.20E-02 |
| ENSDART00000140480 | si:dkey-43f9.1 [Source:ZFIN;Acc:ZDB-GENE-060503-829] | 0.00 | 1.67E-02 |
| ENSDART00000126720 | Uncharacterized protein [Source:UniProtKB/TrEMBL;Acc:E7FG62] | 0.00 | 1.72E-02 |
| ENSDART00000144990 | si:dkey-105i14.1 [Source:ZFIN;Acc:ZDB-GENE-081103-64] | 0.00 | 2.80E-02 |
| ENSDART00000143985 | si:dkey-21o19.8 [Source:ZFIN;Acc:ZDB-GENE-090312-13] | 0.00 | 3.95E-02 |
| ENSDART00000133923 | si:dkey-189d19.1 [Source:ZFIN;Acc:ZDB-GENE-060503-148] | 0.00 | 4.03E-02 |
| ENSDART00000132250 | si:dkeyp-75d2.3 [Source:ZFIN;Acc:ZDB-GENE-070705-249] | 0.00 | 4.03E-02 |

**Table S2. Functional annotation clustering result by DAVID 6.7.**

Functional annotation clustering involved the clustering of terms having similar biological meaning with enrichment indicated by the EASE score or the modified Fisher exact P-value.

| Annotation Cluster 1 | Enrichment Score: 14.610682988310831 |  |  |
| --- | --- | --- | --- |
| Category | Term | Count | PValue |
| KEGG_PATHWAY | dre03010:Ribosome | 27 | 1.93E-24 |
| GOTERM_MF_FAT | GO:0005198~structural molecule activity | 37 | 2.76E-20 |
| GOTERM_MF_FAT | GO:0003735~structural constituent of ribosome | 26 | 8.70E-19 |
| SP_PIR_KEYWORDS | ribosomal protein | 25 | 1.18E-18 |
| GOTERM_CC_FAT | GO:0005840~ribosome | 27 | 1.57E-16 |
| GOTERM_BP_FAT | GO:0006412~translation | 27 | 5.27E-13 |
| GOTERM_CC_FAT | GO:0030529~ribonucleoprotein complex | 28 | 1.44E-12 |
| SP_PIR_KEYWORDS | ribonucleoprotein | 17 | 1.73E-10 |
| GOTERM_CC_FAT | GO:0043232~intracellular non-membrane-bounded organelle | 38 | 2.64E-09 |
| GOTERM_CC_FAT | GO:0043228~non-membrane-bounded organelle | 38 | 2.64E-09 |
|  |  |  |  |
| Annotation Cluster 2 | Enrichment Score: 1.3970443328481448 |  |  |
| Category | Term | Count | PValue |
| INTERPRO | IPR002948:Thiazide-sensitive Na-K-Cl co-transporter | 3 | 0.00142 |
| INTERPRO | IPR004842:Na/K/Cl co-transporter superfamily | 3 | 0.00234 |
| GOTERM_MF_FAT | GO:0015377~cation:chloride symporter activity | 3 | 0.00333 |
| INTERPRO | IPR004841:Amino acid permease-associated region | 4 | 0.00403 |
| GOTERM_BP_FAT | GO:0006821~chloride transport | 4 | 0.0105 |
| GOTERM_MF_FAT | GO:0015296~anion:cation symporter activity | 3 | 0.02019 |
| GOTERM_BP_FAT | GO:0006814~sodium ion transport | 5 | 0.03263 |
| GOTERM_MF_FAT | GO:0015293~symporter activity | 5 | 0.03776 |
| GOTERM_BP_FAT | GO:0015698~inorganic anion transport | 4 | 0.03851 |
| GOTERM_BP_FAT | GO:0015672~monovalent inorganic cation transport | 8 | 0.07171 |
| GOTERM_BP_FAT | GO:0055085~transmembrane transport | 12 | 0.0783 |
| GOTERM_MF_FAT | GO:0015294~solute:cation symporter activity | 4 | 0.0936 |
| GOTERM_MF_FAT | GO:0008509~anion transmembrane transporter activity | 4 | 0.09733 |
| GOTERM_BP_FAT | GO:0006820~anion transport | 4 | 0.10037 |
| GOTERM_BP_FAT | GO:0006812~cation transport | 9 | 0.19999 |
| GOTERM_BP_FAT | GO:0006811~ion transport | 10 | 0.35554 |
| GOTERM_BP_FAT | GO:0030001~metal ion transport | 5 | 0.6061 |
| SP_PIR_KEYWORDS | ion transport | 4 | 0.7193 |
|  |  |  |  |
| Annotation Cluster 3 | Enrichment Score: 1.2476137982166307 |  |  |
| Category | Term | Count | PValue |
| KEGG_PATHWAY | dre00511:Other glycan degradation | 4 | 0.00434 |
| GOTERM_BP_FAT | GO:0019318~hexose metabolic process | 4 | 0.19445 |
| GOTERM_BP_FAT | GO:0005996~monosaccharide metabolic process | 4 | 0.21437 |
|  |  |  |  |
| Annotation Cluster 4 | Enrichment Score: 1.130153757748044 |  |  |
| Category | Term | Count | PValue |
| GOTERM_BP_FAT | GO:0030203~glycosaminoglycan metabolic process | 3 | 0.04602 |
| GOTERM_BP_FAT | GO:0006022~aminoglycan metabolic process | 3 | 0.06086 |
| GOTERM_BP_FAT | GO:0005976~polysaccharide metabolic process | 3 | 0.14532 |
|  |  |  |  |
| Annotation Cluster 5 | Enrichment Score: 0.8216651846191793 |  |  |
| Category | Term | Count | PValue |
| GOTERM_MF_FAT | GO:0004857~enzyme inhibitor activity | 5 | 0.11027 |
| GOTERM_MF_FAT | GO:0004866~endopeptidase inhibitor activity | 4 | 0.13771 |
| GOTERM_MF_FAT | GO:0030414~peptidase inhibitor activity | 4 | 0.17805 |
| GOTERM_MF_FAT | GO:0004867~serine-type endopeptidase inhibitor activity | 3 | 0.19115 |
|  |  |  |  |
| Annotation Cluster 6 | Enrichment Score: 0.6892614556143813 |  |  |
| Category | Term | Count | PValue |
| GOTERM_BP_FAT | GO:0010605~negative regulation of macromolecule metabolic process | 4 | 0.12934 |
| GOTERM_BP_FAT | GO:0010558~negative regulation of macromolecule biosynthetic process | 3 | 0.23591 |
| GOTERM_BP_FAT | GO:0031327~negative regulation of cellular biosynthetic process | 3 | 0.23591 |
| GOTERM_BP_FAT | GO:0009890~negative regulation of biosynthetic process | 3 | 0.24307 |
|  |  |  |  |
| Annotation Cluster 7 | Enrichment Score: 0.6638221944133489 |  |  |
| Category | Term | Count | PValue |
| GOTERM_MF_FAT | GO:0005201~extracellular matrix structural constituent | 3 | 0.03915 |
| GOTERM_CC_FAT | GO:0005578~proteinaceous extracellular matrix | 4 | 0.27663 |
| GOTERM_CC_FAT | GO:0031012~extracellular matrix | 4 | 0.29506 |
| GOTERM_CC_FAT | GO:0005576~extracellular region | 11 | 0.36838 |
| GOTERM_CC_FAT | GO:0044421~extracellular region part | 5 | 0.40741 |
|  |  |  |  |
| Annotation Cluster 8 | Enrichment Score: 0.6592661500279597 |  |  |
| Category | Term | Count | PValue |
| GOTERM_MF_FAT | GO:0004896~cytokine receptor activity | 3 | 0.07305 |
| GOTERM_MF_FAT | GO:0019955~cytokine binding | 3 | 0.14468 |
| SP_PIR_KEYWORDS | receptor | 7 | 0.99581 |
|  |  |  |  |
| Annotation Cluster 9 | Enrichment Score: 0.6110112125652182 |  |  |
| Category | Term | Count | PValue |
| INTERPRO | IPR002957:Keratin, type I | 3 | 0.04176 |
| INTERPRO | IPR001664:Intermediate filament protein | 3 | 0.13894 |
| INTERPRO | IPR016044:Filament | 3 | 0.13894 |
| SP_PIR_KEYWORDS | Intermediate filament | 3 | 0.17049 |
| GOTERM_CC_FAT | GO:0045111~intermediate filament cytoskeleton | 3 | 0.24096 |
| GOTERM_CC_FAT | GO:0005882~intermediate filament | 3 | 0.24096 |
| GOTERM_CC_FAT | GO:0044430~cytoskeletal part | 5 | 0.66525 |
| SP_PIR_KEYWORDS | coiled coil | 6 | 0.74395 |
| GOTERM_CC_FAT | GO:0005856~cytoskeleton | 6 | 0.80243 |
|  |  |  |  |
| Annotation Cluster 10 | Enrichment Score: 0.5988687934339267 |  |  |
| Category | Term | Count | PValue |
| GOTERM_MF_FAT | GO:0015078~hydrogen ion transmembrane transporter activity | 5 | 0.03776 |
| KEGG_PATHWAY | dre00190:Oxidative phosphorylation | 7 | 0.05028 |
| GOTERM_MF_FAT | GO:0015077~monovalent inorganic cation transmembrane transporter activity | 5 | 0.0553 |
| GOTERM_CC_FAT | GO:0033177~proton-transporting two-sector ATPase complex, proton-transporting domain | 3 | 0.07871 |
| GOTERM_BP_FAT | GO:0044271~nitrogen compound biosynthetic process | 8 | 0.12453 |
| GOTERM_MF_FAT | GO:0022890~inorganic cation transmembrane transporter activity | 5 | 0.15652 |
| GOTERM_BP_FAT | GO:0015986~ATP synthesis coupled proton transport | 3 | 0.22164 |
| GOTERM_BP_FAT | GO:0015985~energy coupled proton transport, down electrochemical gradient | 3 | 0.22164 |
| GOTERM_BP_FAT | GO:0009145~purine nucleoside triphosphate biosynthetic process | 4 | 0.22449 |
| GOTERM_BP_FAT | GO:0009142~nucleoside triphosphate biosynthetic process | 4 | 0.22449 |
| GOTERM_BP_FAT | GO:0009206~purine ribonucleoside triphosphate biosynthetic process | 4 | 0.22449 |
| GOTERM_BP_FAT | GO:0009201~ribonucleoside triphosphate biosynthetic process | 4 | 0.22449 |
| GOTERM_BP_FAT | GO:0009144~purine nucleoside triphosphate metabolic process | 4 | 0.22958 |
| GOTERM_BP_FAT | GO:0009141~nucleoside triphosphate metabolic process | 4 | 0.22958 |
| GOTERM_BP_FAT | GO:0009199~ribonucleoside triphosphate metabolic process | 4 | 0.22958 |
| GOTERM_BP_FAT | GO:0009205~purine ribonucleoside triphosphate metabolic process | 4 | 0.22958 |
| GOTERM_BP_FAT | GO:0006818~hydrogen transport | 3 | 0.23591 |
| GOTERM_BP_FAT | GO:0015992~proton transport | 3 | 0.23591 |
| GOTERM_BP_FAT | GO:0034220~ion transmembrane transport | 3 | 0.23591 |
| GOTERM_CC_FAT | GO:0016469~proton-transporting two-sector ATPase complex | 3 | 0.28271 |
| GOTERM_BP_FAT | GO:0009165~nucleotide biosynthetic process | 5 | 0.2834 |
| GOTERM_BP_FAT | GO:0006119~oxidative phosphorylation | 3 | 0.30758 |
| GOTERM_BP_FAT | GO:0009152~purine ribonucleotide biosynthetic process | 4 | 0.31324 |
| GOTERM_BP_FAT | GO:0009150~purine ribonucleotide metabolic process | 4 | 0.31854 |
| GOTERM_BP_FAT | GO:0034404~nucleobase, nucleoside and nucleotide biosynthetic process | 5 | 0.32701 |
| GOTERM_BP_FAT | GO:0034654~nucleobase, nucleoside, nucleotide and nucleic acid biosynthetic process | 5 | 0.32701 |
| GOTERM_BP_FAT | GO:0009260~ribonucleotide biosynthetic process | 4 | 0.33444 |
| GOTERM_BP_FAT | GO:0009259~ribonucleotide metabolic process | 4 | 0.33974 |
| GOTERM_BP_FAT | GO:0006164~purine nucleotide biosynthetic process | 4 | 0.3662 |
| GOTERM_BP_FAT | GO:0006163~purine nucleotide metabolic process | 4 | 0.38199 |
| GOTERM_BP_FAT | GO:0006754~ATP biosynthetic process | 3 | 0.43871 |
| GOTERM_BP_FAT | GO:0046034~ATP metabolic process | 3 | 0.44527 |
| GOTERM_BP_FAT | GO:0006091~generation of precursor metabolites and energy | 4 | 0.48914 |
| GOTERM_BP_FAT | GO:0016310~phosphorylation | 6 | 0.95947 |
| GOTERM_BP_FAT | GO:0006796~phosphate metabolic process | 6 | 0.99125 |
| GOTERM_BP_FAT | GO:0006793~phosphorus metabolic process | 6 | 0.99125 |
|  |  |  |  |
| Annotation Cluster 11 | Enrichment Score: 0.49097078854855186 |  |  |
| Category | Term | Count | PValue |
| INTERPRO | IPR017974:Claudin, conserved site | 3 | 0.06571 |
| INTERPRO | IPR006187:Claudin | 3 | 0.09796 |
| SP_PIR_KEYWORDS | Tight junction | 3 | 0.10408 |
| INTERPRO | IPR004031:PMP-22/EMP/MP20/Claudin | 3 | 0.16613 |
| GOTERM_CC_FAT | GO:0070160~occluding junction | 3 | 0.24929 |
| GOTERM_CC_FAT | GO:0005923~tight junction | 3 | 0.24929 |
| GOTERM_CC_FAT | GO:0043296~apical junction complex | 3 | 0.28271 |
| GOTERM_CC_FAT | GO:0016327~apicolateral plasma membrane | 3 | 0.28271 |
| GOTERM_CC_FAT | GO:0005911~cell-cell junction | 3 | 0.55166 |
| SP_PIR_KEYWORDS | cell junction | 3 | 0.60443 |
| GOTERM_CC_FAT | GO:0030054~cell junction | 3 | 0.82747 |
| SP_PIR_KEYWORDS | cell membrane | 4 | 0.87946 |
| GOTERM_CC_FAT | GO:0044459~plasma membrane part | 3 | 0.99823 |
| GOTERM_CC_FAT | GO:0005886~plasma membrane | 5 | 0.99905 |
|  |  |  |  |
| Annotation Cluster 12 | Enrichment Score: 0.4687828145967223 |  |  |
| Category | Term | Count | PValue |
| INTERPRO | IPR002401:Cytochrome P450, E-class, group I | 3 | 0.21122 |
| INTERPRO | IPR017973:Cytochrome P450, C-terminal region | 3 | 0.22269 |
| INTERPRO | IPR017972:Cytochrome P450, conserved site | 3 | 0.23998 |
| INTERPRO | IPR001128:Cytochrome P450 | 3 | 0.25733 |
| GOTERM_MF_FAT | GO:0020037~heme binding | 4 | 0.30181 |
| GOTERM_MF_FAT | GO:0046906~tetrapyrrole binding | 4 | 0.32747 |
| SP_PIR_KEYWORDS | iron | 5 | 0.34406 |
| COG_ONTOLOGY | Secondary metabolites biosynthesis, transport, and catabolism | 3 | 0.37287 |
| SP_PIR_KEYWORDS | Monooxygenase | 3 | 0.37912 |
| GOTERM_MF_FAT | GO:0005506~iron ion binding | 6 | 0.44038 |
| SP_PIR_KEYWORDS | heme | 3 | 0.46504 |
| GOTERM_MF_FAT | GO:0009055~electron carrier activity | 3 | 0.82857 |
|  |  |  |  |
| Annotation Cluster 13 | Enrichment Score: 0.4383084039361041 |  |  |
| Category | Term | Count | PValue |
| GOTERM_BP_FAT | GO:0034622~cellular macromolecular complex assembly | 4 | 0.26057 |
| GOTERM_BP_FAT | GO:0034621~cellular macromolecular complex subunit organization | 4 | 0.30794 |
| GOTERM_BP_FAT | GO:0065003~macromolecular complex assembly | 4 | 0.45428 |
| GOTERM_BP_FAT | GO:0043933~macromolecular complex subunit organization | 4 | 0.48423 |
|  |  |  |  |
| Annotation Cluster 14 | Enrichment Score: 0.25527724033679783 |  |  |
| Category | Term | Count | PValue |
| SMART | SM00060:FN3 | 3 | 0.43625 |
| INTERPRO | IPR003961:Fibronectin, type III | 3 | 0.62093 |
| INTERPRO | IPR008957:Fibronectin, type III-like fold | 3 | 0.63298 |
|  |  |  |  |
| Annotation Cluster 15 | Enrichment Score: 0.24682407429068964 |  |  |
| Category | Term | Count | PValue |
| SP_PIR_KEYWORDS | mitochondrion inner membrane | 3 | 0.2551 |
| GOTERM_CC_FAT | GO:0005743~mitochondrial inner membrane | 4 | 0.34448 |
| GOTERM_CC_FAT | GO:0019866~organelle inner membrane | 4 | 0.363 |
| SP_PIR_KEYWORDS | mitochondrion | 4 | 0.48625 |
| GOTERM_CC_FAT | GO:0031966~mitochondrial membrane | 4 | 0.52262 |
| GOTERM_CC_FAT | GO:0005740~mitochondrial envelope | 4 | 0.58146 |
| GOTERM_CC_FAT | GO:0044429~mitochondrial part | 4 | 0.68057 |
| UP_SEQ_FEATURE | transmembrane region | 8 | 0.73876 |
| GOTERM_CC_FAT | GO:0031967~organelle envelope | 4 | 0.77161 |
| GOTERM_CC_FAT | GO:0031975~envelope | 4 | 0.77491 |
| GOTERM_CC_FAT | GO:0005739~mitochondrion | 5 | 0.81492 |
| GOTERM_CC_FAT | GO:0031090~organelle membrane | 4 | 0.94542 |
|  |  |  |  |
| Annotation Cluster 16 | Enrichment Score: 0.2315202897311807 |  |  |
| Category | Term | Count | PValue |
| SMART | SM00320:WD40 | 4 | 0.32108 |
| INTERPRO | IPR001680:WD40 repeat | 4 | 0.53775 |
| SP_PIR_KEYWORDS | wd repeat | 4 | 0.5928 |
| INTERPRO | IPR015943:WD40/YVTN repeat-like | 4 | 0.62284 |
| INTERPRO | IPR019782:WD40 repeat 2 | 3 | 0.70246 |
| INTERPRO | IPR017986:WD40 repeat, region | 3 | 0.72829 |
| INTERPRO | IPR019781:WD40 repeat, subgroup | 3 | 0.73444 |
|  |  |  |  |
| Annotation Cluster 17 | Enrichment Score: 0.1639426309099861 |  |  |
| Category | Term | Count | PValue |
| GOTERM_CC_FAT | GO:0044427~chromosomal part | 3 | 0.58417 |
| GOTERM_BP_FAT | GO:0051276~chromosome organization | 3 | 0.74221 |
| GOTERM_CC_FAT | GO:0005694~chromosome | 3 | 0.7432 |
|  |  |  |  |
| Annotation Cluster 18 | Enrichment Score: 0.14040081017844916 |  |  |
| Category | Term | Count | PValue |
| KEGG_PATHWAY | dre04540:Gap junction | 4 | 0.33476 |
| GOTERM_MF_FAT | GO:0003924~GTPase activity | 3 | 0.34304 |
| GOTERM_MF_FAT | GO:0005525~GTP binding | 8 | 0.41045 |
| GOTERM_MF_FAT | GO:0032561~guanyl ribonucleotide binding | 8 | 0.41972 |
| GOTERM_MF_FAT | GO:0019001~guanyl nucleotide binding | 8 | 0.42281 |
| SP_PIR_KEYWORDS | gtp-binding | 5 | 0.70593 |
| SP_PIR_KEYWORDS | nucleotide-binding | 13 | 0.91159 |
| GOTERM_MF_FAT | GO:0032555~purine ribonucleotide binding | 18 | 0.94229 |
| GOTERM_MF_FAT | GO:0032553~ribonucleotide binding | 18 | 0.94229 |
| SP_PIR_KEYWORDS | atp-binding | 8 | 0.95851 |
| GOTERM_MF_FAT | GO:0017076~purine nucleotide binding | 18 | 0.96089 |
| GOTERM_MF_FAT | GO:0000166~nucleotide binding | 21 | 0.97942 |
| GOTERM_MF_FAT | GO:0005524~ATP binding | 11 | 0.9864 |
| GOTERM_MF_FAT | GO:0032559~adenyl ribonucleotide binding | 11 | 0.98686 |
| GOTERM_MF_FAT | GO:0030554~adenyl nucleotide binding | 11 | 0.99222 |
| GOTERM_MF_FAT | GO:0001883~purine nucleoside binding | 11 | 0.99249 |
| GOTERM_MF_FAT | GO:0001882~nucleoside binding | 11 | 0.99293 |
|  |  |  |  |
| Annotation Cluster 19 | Enrichment Score: 0.13316995644441224 |  |  |
| Category | Term | Count | PValue |
| GOTERM_MF_FAT | GO:0017171~serine hydrolase activity | 3 | 0.6225 |
| GOTERM_MF_FAT | GO:0008236~serine-type peptidase activity | 3 | 0.6225 |
| GOTERM_MF_FAT | GO:0004175~endopeptidase activity | 5 | 0.68864 |
| GOTERM_MF_FAT | GO:0070011~peptidase activity, acting on L-amino acid peptides | 6 | 0.82747 |
| GOTERM_BP_FAT | GO:0006508~proteolysis | 8 | 0.83632 |
| GOTERM_MF_FAT | GO:0008233~peptidase activity | 6 | 0.86017 |
|  |  |  |  |
| Annotation Cluster 20 | Enrichment Score: 0.11883194426030122 |  |  |
| Category | Term | Count | PValue |
| INTERPRO | IPR018248:EF hand | 3 | 0.56523 |
| INTERPRO | IPR018247:EF-HAND 1 | 3 | 0.7743 |
| INTERPRO | IPR018249:EF-HAND 2 | 3 | 0.77955 |
| GOTERM_MF_FAT | GO:0005509~calcium ion binding | 7 | 0.79819 |
| INTERPRO | IPR011992:EF-Hand type | 3 | 0.80187 |
| SP_PIR_KEYWORDS | calcium | 4 | 0.8868 |
|  |  |  |  |
| Annotation Cluster 21 | Enrichment Score: 0.1011140331076084 |  |  |
| Category | Term | Count | PValue |
| INTERPRO | IPR017907:Zinc finger, RING-type, conserved site | 3 | 0.6853 |
| SMART | SM00184:RING | 3 | 0.72635 |
| INTERPRO | IPR001841:Zinc finger, RING-type | 3 | 0.87707 |
| SP_PIR_KEYWORDS | zinc-finger | 5 | 0.90257 |
|  |  |  |  |
| Annotation Cluster 22 | Enrichment Score: 0.06796127740693311 |  |  |
| Category | Term | Count | PValue |
| UP_SEQ_FEATURE | topological domain:Extracellular | 3 | 0.76392 |
| UP_SEQ_FEATURE | glycosylation site:N-linked (GlcNAc...) | 4 | 0.83719 |
| UP_SEQ_FEATURE | topological domain:Cytoplasmic | 3 | 0.91315 |
| SP_PIR_KEYWORDS | glycoprotein | 4 | 0.91568 |
|  |  |  |  |
| Annotation Cluster 23 | Enrichment Score: 0.0670917016983671 |  |  |
| Category | Term | Count | PValue |
| INTERPRO | IPR013087:Zinc finger, C2H2-type/integrase, DNA-binding | 4 | 0.77013 |
| SMART | SM00355:ZnF_C2H2 | 4 | 0.79177 |
| INTERPRO | IPR015880:Zinc finger, C2H2-like | 4 | 0.93573 |
| INTERPRO | IPR007087:Zinc finger, C2H2-type | 4 | 0.94476 |
|  |  |  |  |
| Annotation Cluster 24 | Enrichment Score: 0.06492702557016887 |  |  |
| Category | Term | Count | PValue |
| INTERPRO | IPR011991:Winged helix repressor DNA-binding | 5 | 0.25947 |
| SP_PIR_KEYWORDS | Transcription | 8 | 0.77504 |
| SP_PIR_KEYWORDS | transcription regulation | 8 | 0.78573 |
| GOTERM_MF_FAT | GO:0043565~sequence-specific DNA binding | 6 | 0.95568 |
| SP_PIR_KEYWORDS | dna-binding | 7 | 0.98042 |
| GOTERM_MF_FAT | GO:0003700~transcription factor activity | 6 | 0.99156 |
| GOTERM_BP_FAT | GO:0006355~regulation of transcription, DNA-dependent | 8 | 0.994 |
| GOTERM_BP_FAT | GO:0051252~regulation of RNA metabolic process | 8 | 0.9944 |
| SP_PIR_KEYWORDS | nucleus | 12 | 0.99581 |
| GOTERM_BP_FAT | GO:0045449~regulation of transcription | 11 | 0.99709 |
| GOTERM_BP_FAT | GO:0006350~transcription | 4 | 0.99737 |
| GOTERM_MF_FAT | GO:0003677~DNA binding | 11 | 0.99778 |
| GOTERM_MF_FAT | GO:0030528~transcription regulator activity | 7 | 0.99878 |
|  |  |  |  |
| Annotation Cluster 25 | Enrichment Score: 0.03859414416019536 |  |  |
| Category | Term | Count | PValue |
| UP_SEQ_FEATURE | transmembrane region | 8 | 0.73876 |
| SP_PIR_KEYWORDS | transport | 8 | 0.83651 |
| SP_PIR_KEYWORDS | membrane | 19 | 0.96723 |
| SP_PIR_KEYWORDS | transmembrane | 20 | 0.98163 |
| GOTERM_CC_FAT | GO:0016021~integral to membrane | 26 | 0.99998 |
| GOTERM_CC_FAT | GO:0031224~intrinsic to membrane | 26 | 0.99999 |
|  |  |  |  |
| Annotation Cluster 26 | Enrichment Score: 0.015458805063245488 |  |  |
| Category | Term | Count | PValue |
| SP_PIR_KEYWORDS | metal-binding | 15 | 0.86467 |
| SP_PIR_KEYWORDS | zinc | 9 | 0.9657 |
| GOTERM_MF_FAT | GO:0046914~transition metal ion binding | 22 | 0.98074 |
| GOTERM_MF_FAT | GO:0043169~cation binding | 32 | 0.98152 |
| GOTERM_MF_FAT | GO:0043167~ion binding | 32 | 0.98167 |
| GOTERM_MF_FAT | GO:0046872~metal ion binding | 30 | 0.99177 |
| GOTERM_MF_FAT | GO:0008270~zinc ion binding | 15 | 0.99602 |
|  |  |  |  |
| Annotation Cluster 27 | Enrichment Score: 0.010645088252571001 |  |  |
| Category | Term | Count | PValue |
| INTERPRO | IPR017442:Serine/threonine protein kinase-related | 3 | 0.94887 |
| SP_PIR_KEYWORDS | atp-binding | 8 | 0.95851 |
| GOTERM_BP_FAT | GO:0016310~phosphorylation | 6 | 0.95947 |
| INTERPRO | IPR017441:Protein kinase, ATP binding site | 3 | 0.96136 |
| GOTERM_MF_FAT | GO:0004674~protein serine/threonine kinase activity | 3 | 0.97258 |
| INTERPRO | IPR000719:Protein kinase, core | 3 | 0.982 |
| GOTERM_BP_FAT | GO:0006796~phosphate metabolic process | 6 | 0.99125 |
| GOTERM_BP_FAT | GO:0006793~phosphorus metabolic process | 6 | 0.99125 |
| GOTERM_MF_FAT | GO:0004672~protein kinase activity | 3 | 0.99665 |
| GOTERM_BP_FAT | GO:0006468~protein amino acid phosphorylation | 3 | 0.99743 |

**Table S3. Functional annotation chart by DAVID 6.7.**

The functional annotation chart provides the enriched terms separately as associated with the list of differentially expressed genes.

| Category | Term | Count | PValue |
| --- | --- | --- | --- |
| KEGG_PATHWAY | dre03010:Ribosome | 27 | 1.93E-24 |
| GOTERM_MF_FAT | GO:0005198~structural molecule activity | 37 | 2.76E-20 |
| GOTERM_MF_FAT | GO:0003735~structural constituent of ribosome | 26 | 8.70E-19 |
| SP_PIR_KEYWORDS | ribosomal protein | 25 | 1.18E-18 |
| GOTERM_CC_FAT | GO:0005840~ribosome | 27 | 1.57E-16 |
| GOTERM_BP_FAT | GO:0006412~translation | 27 | 5.27E-13 |
| GOTERM_CC_FAT | GO:0030529~ribonucleoprotein complex | 28 | 1.44E-12 |
| SP_PIR_KEYWORDS | ribonucleoprotein | 17 | 1.73E-10 |
| GOTERM_CC_FAT | GO:0043228~non-membrane-bounded organelle | 38 | 2.64E-09 |
| GOTERM_CC_FAT | GO:0043232~intracellular non-membrane-bounded organelle | 38 | 2.64E-09 |
| INTERPRO | IPR009311:Interferon-induced 6-16 | 4 | 7.19E-05 |
| INTERPRO | IPR002948:Thiazide-sensitive Na-K-Cl co-transporter | 3 | 0.001419055 |
| GOTERM_MF_FAT | GO:0019843~rRNA binding | 4 | 0.001991167 |
| INTERPRO | IPR004842:Na/K/Cl co-transporter superfamily | 3 | 0.002340843 |
| GOTERM_MF_FAT | GO:0015377~cation:chloride symporter activity | 3 | 0.003332241 |
| INTERPRO | IPR004841:Amino acid permease-associated region | 4 | 0.004025301 |
| KEGG_PATHWAY | dre00511:Other glycan degradation | 4 | 0.004337027 |
| GOTERM_BP_FAT | GO:0006821~chloride transport | 4 | 0.010499404 |
| GOTERM_CC_FAT | GO:0033279~ribosomal subunit | 4 | 0.01551361 |
| GOTERM_MF_FAT | GO:0015296~anion:cation symporter activity | 3 | 0.02018772 |
| GOTERM_CC_FAT | GO:0015935~small ribosomal subunit | 3 | 0.023787124 |
| SP_PIR_KEYWORDS | oxidoreductase | 11 | 0.028574158 |
| INTERPRO | IPR003204:Cytochrome c oxidase, subunit Va | 2 | 0.030916313 |
| INTERPRO | IPR000892:Ribosomal protein S26e | 2 | 0.030916313 |
| GOTERM_BP_FAT | GO:0006814~sodium ion transport | 5 | 0.032630777 |
| SMART | SM00812:Alpha_L_fucos | 2 | 0.032661078 |
| GOTERM_MF_FAT | GO:0019961~interferon binding | 2 | 0.03697354 |
| GOTERM_MF_FAT | GO:0004904~interferon receptor activity | 2 | 0.03697354 |
| GOTERM_BP_FAT | GO:0006414~translational elongation | 3 | 0.037000309 |
| GOTERM_MF_FAT | GO:0015293~symporter activity | 5 | 0.037758535 |
| GOTERM_MF_FAT | GO:0015078~hydrogen ion transmembrane transporter activity | 5 | 0.037758535 |
| GOTERM_BP_FAT | GO:0015698~inorganic anion transport | 4 | 0.038510963 |
| GOTERM_MF_FAT | GO:0005201~extracellular matrix structural constituent | 3 | 0.039152281 |
| INTERPRO | IPR002957:Keratin, type I | 3 | 0.041759126 |
| PIR_SUPERFAMILY | PIRSF000275:cytochrome-c oxidase chain Va | 2 | 0.044738142 |
| INTERPRO | IPR000086:NUDIX hydrolase domain | 3 | 0.045474242 |
| INTERPRO | IPR018526:Glycoside hydrolase, family 29, conserved site | 2 | 0.046016026 |
| INTERPRO | IPR000933:Glycoside hydrolase, family 29 | 2 | 0.046016026 |
| INTERPRO | IPR016286:Glycoside hydrolase, family 29, subgroup | 2 | 0.046016026 |
| GOTERM_BP_FAT | GO:0030203~glycosaminoglycan metabolic process | 3 | 0.046016321 |
| KEGG_PATHWAY | dre00190:Oxidative phosphorylation | 7 | 0.050276549 |
| GOTERM_BP_FAT | GO:0006720~isoprenoid metabolic process | 3 | 0.050795794 |
| GOTERM_MF_FAT | GO:0015928~fucosidase activity | 2 | 0.054948009 |
| GOTERM_MF_FAT | GO:0004560~alpha-L-fucosidase activity | 2 | 0.054948009 |
| GOTERM_MF_FAT | GO:0015077~monovalent inorganic cation transmembrane transporter activity | 5 | 0.05529769 |
| GOTERM_BP_FAT | GO:0006004~fucose metabolic process | 2 | 0.056655704 |
| GOTERM_BP_FAT | GO:0002764~immune response-regulating signal transduction | 2 | 0.056655704 |
| GOTERM_BP_FAT | GO:0002757~immune response-activating signal transduction | 2 | 0.056655704 |
| GOTERM_BP_FAT | GO:0006022~aminoglycan metabolic process | 3 | 0.060855442 |
| INTERPRO | IPR001813:Ribosomal protein 60S | 2 | 0.060881683 |
| INTERPRO | IPR017974:Claudin, conserved site | 3 | 0.065713592 |
| PIR_SUPERFAMILY | PIRSF001092:Alpha-L-fucosidase | 2 | 0.066359295 |
| GOTERM_BP_FAT | GO:0015672~monovalent inorganic cation transport | 8 | 0.071708757 |
| GOTERM_MF_FAT | GO:0004896~cytokine receptor activity | 3 | 0.073049293 |
| GOTERM_BP_FAT | GO:0002253~activation of immune response | 2 | 0.074823816 |
| INTERPRO | IPR013612:Amino acid permease N-terminal | 2 | 0.075516893 |
| INTERPRO | IPR011331:Ribosomal protein L37ae/L37e, core | 2 | 0.075516893 |
| GOTERM_BP_FAT | GO:0055085~transmembrane transport | 12 | 0.078298922 |
| GOTERM_CC_FAT | GO:0033177~proton-transporting two-sector ATPase complex, proton-transporting domain | 3 | 0.078705616 |
| SP_PIR_KEYWORDS | cf(0) | 2 | 0.085618258 |
| GOTERM_BP_FAT | GO:0050778~positive regulation of immune response | 2 | 0.092644664 |
| GOTERM_BP_FAT | GO:0002684~positive regulation of immune system process | 2 | 0.092644664 |
| GOTERM_MF_FAT | GO:0015294~solute:cation symporter activity | 4 | 0.093598545 |
| GOTERM_MF_FAT | GO:0008509~anion transmembrane transporter activity | 4 | 0.09732908 |
| INTERPRO | IPR006187:Claudin | 3 | 0.097956036 |

**Table S4. Gene functional classification result by DAVID 6.7.**

Gene functional classification involves a gene-centric annotation approach classifying the differentially expressed genes into functional related gene groups on basis of enrichment scores.

| Gene Group 1 | Enrichment Score: 14.610682988310831 |
| --- | --- |
| ENSEMBL_GENE_ID | Gene Name |
| ENSDARG00000025073 | ribosomal protein L18a |
| ENSDARG00000051783 | ribosomal protein, large, P0 |
| ENSDARG00000034291 | ribosomal protein L37; hypothetical LOC100000999 |
| ENSDARG00000042389 | zgc:171772 |
| ENSDARG00000034897 | ribosomal protein S10 |
| ENSDARG00000030602 | ribosomal protein S19 |
| ENSDARG00000043509 | ribosomal protein L11 |
| ENSDARG00000057556 | zgc:65996 |
| ENSDARG00000009285 | ribosomal protein L15 |
| ENSDARG00000015490 | ribosomal protein L24 |
| ENSDARG00000037071 | ribosomal protein S26 |
| ENSDARG00000014867 | ribosomal protein L8 |
| ENSDARG00000036875 | ribosomal protein S12 |
| ENSDARG00000037350 | similar to ribosomal protein L9; ribosomal protein L9 |
| ENSDARG00000011201 | ribosomal protein, large P2, like |
| ENSDARG00000035692 | ribosomal protein S3A |
| ENSDARG00000053457 | similar to ribosomal protein L23; ribosomal protein L23 |
| ENSDARG00000053058 | ribosomal protein S11 |
| ENSDARG00000023298, | zgc:73262; zgc:109888 |
| ENSDARG00000055475 |  |
| ENSDARG00000070849 | ribosomal protein S15 |
| ENSDARG00000030408 | ribosomal protein S26, like |
| ENSDARG00000041435 | ubiquitin A-52 residue ribosomal protein fusion product 1 |
| ENSDARG00000020197 | ribosomal protein L5a |
| ENSDARG00000077291 | ribosomal protein S2 |
| ENSDARG00000046119 | ribosomal protein S3 |
| ENSDARG00000013012 | ribosomal protein L36 |
| ENSDARG00000035871 | ribosomal protein L30 |
|  |  |
| Gene Group 2 | Enrichment Score: 1.3062838558522554 |
| ENSEMBL_GENE_ID | Gene Name |
| ENSDARG00000013743 | solute carrier family 12 (sodium/potassium/chloride transporters), member 10.1;  solute carrier family 12, member 2-like |
| ENSDARG00000053853 | solute carrier family 13 (sodium-dependent dicarboxylate transporter), member 2 |
| ENSDARG00000055253 | similar to Solute carrier family 12 member 3 (Thiazide-sensitive sodium-chloride cotransporter)  (Na-Cl symporter); |
|  | slc12a10.3 solute carrier family 12 (sodium/potassium/chloride transporters), member 10.3 |
| ENSDARG00000013855 | solute carrier family 12 (sodium/chloride transporters), member 3 |
|  |  |
| Gene Group 3 | Enrichment Score: 0.2209577159354666 |
| ENSEMBL_GENE_ID | Gene Name |
| ENSDARG00000045019 | zgc:85939 |
| ENSDARG00000041619 | guanine nucleotide binding protein (G protein), beta polypeptide 2-like 1 |
| ENSDARG00000021557 | wdr45 like |
| ENSDARG00000075883 | BUB3 budding uninhibited by benzimidazoles 3 homolog (yeast) |
|  |  |
| Gene Group 4 | Enrichment Score: 0.09254709428745779 |
| ENSEMBL_GENE_ID | Gene Name |
| ENSDARG00000041411 | RAD51 homolog (RecA homolog, E. coli) (S. cerevisiae) |
| ENSDARG00000035178 | guanine nucleotide binding protein (G protein), alpha 14 |
| ENSDARG00000031164 | tubulin, alpha 8 like 2 |
| ENSDARG00000040984 | heat shock protein 13 |
| ENSDARG00000078093 | zgc:172065; hypothetical LOC100001153 |
| ENSDARG00000056443 | zgc:152753 |
| ENSDARG00000055385 | zgc:110560; hypothetical protein LOC100150958 |
| ENSDARG00000030644 | guanine nucleotide binding protein (G protein), alpha inhibiting activity polypeptide 3 |
| ENSDARG00000002210 | zgc:92836 |
| ENSDARG00000052900 | zgc:153642 |
| ENSDARG00000074873 | similar to Serine/threonine-protein kinase Pim-3; si:dkey-108d22.5 |
| ENSDARG00000014373 | vasa homolog |
| ENSDARG00000015134 | similar to calcium/calmodulin-dependent protein kinase kinase 1, alpha; zgc:194737 |
| ENSDARG00000002344 | zgc:55461; zgc:123194; zgc:153264; zgc:123292; tubulin, beta 2c; zgc:153426 |
|  |  |
| Gene Group 5 | Enrichment Score: 0.023363315209822887 |
| ENSEMBL_GENE_ID | Gene Name |
| ENSDARG00000056407 | interferon regulatory factor 8 |
| ENSDARG00000070769 | forkhead box G1 |
| ENSDARG00000058133 | forkhead box D1 |
| ENSDARG00000079406 | homeo box C11a; homeo box C11b |
| ENSDARG00000069337 | Mediator of RNA polymerase II transcription subunit 11 |
| ENSDARG00000040253 | one cut domain, family member, like |
| ENSDARG00000043531 | v-jun sarcoma virus 17 oncogene homolog (avian) |
|  |  |
| Gene Group 6 | Enrichment Score: 0.015458805063245488 |
| ENSEMBL_GENE_ID | Gene Name |
| ENSDARG00000070864 | B-cell CLL/lymphoma 6 (zinc finger protein 51) |
| ENSDARG00000014794 | zgc:92453 |
| ENSDARG00000008218 | zgc:77303 |
| ENSDARG00000038006 | odd-skipped related 2 (Drosophila) |
| ENSDARG00000028476 | zgc:65779; hypothetical LOC791614 |
| ENSDARG00000071558 | zgc:154176 |
| ENSDARG00000043323 | ligand of numb-protein X 1 |
| ENSDARG00000001897 | zgc:110815 |
| ENSDARG00000013279 | zgc:153635 |
| ENSDARG00000023298 | zgc:73262; zgc:109888 |
| ENSDARG00000055475 |  |
| ENSDARG00000021677 | similar to PHD finger protein 6 |
| ENSDARG00000056907 | zgc:173949; hypothetical protein LOC100006493; similar to retinoblastoma binding protein 6; similar to retinoblastoma-binding protein 6 |
|  |  |
| Gene Group 7 | Enrichment Score: 0.005634640019764196 |
| ENSEMBL_GENE_ID | Gene Name |
| ENSDARG00000044990 | zgc:65811 |
| ENSDARG00000027065 | zgc:165543; similar to Sodium-coupled neutral amino acid transporter 3 (Na(+)-coupled neutral amino acid transporter 3) |
|  | (System N amino acid transporter 1) (N-system amino acid transporter 1) (Solute carrier family 38 member 3) |
| ENSDARG00000076899 | solute carrier family 2 (facilitated glucose transporter), member 13a |
| ENSDARG00000055307 | synaptophysin-like 2a |
| ENSDARG00000059824 | hypothetical LOC564868; zgc:153102 |
| ENSDARG00000019137 | translocating chain-associating membrane protein 1 |

## Supplementary Figures

**
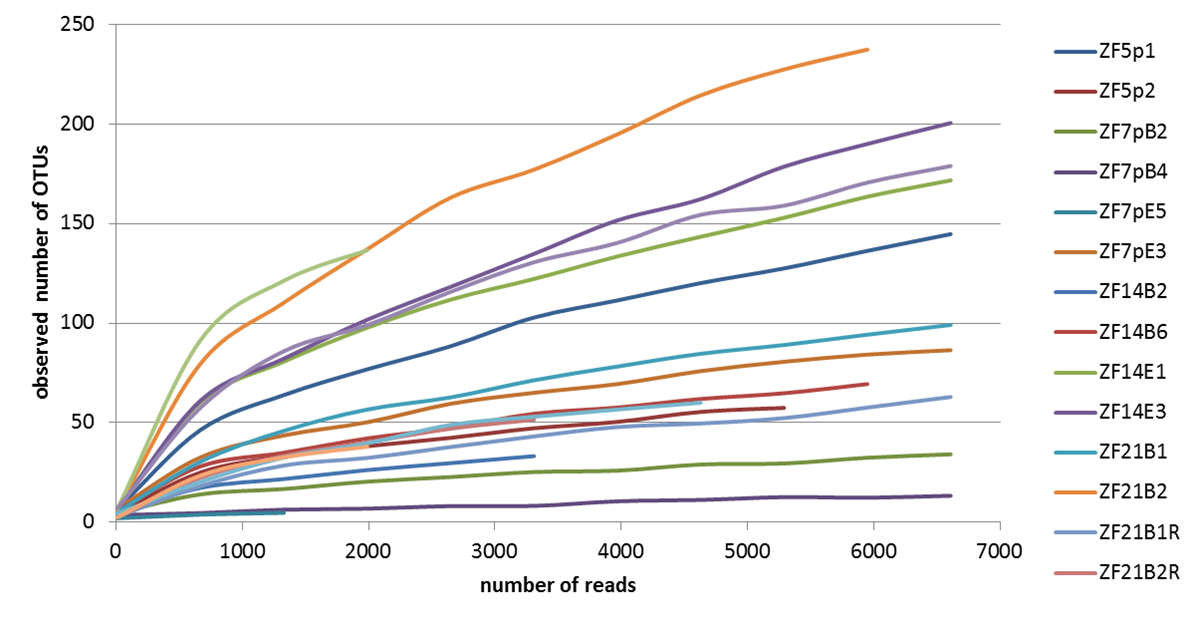
**

**Fig. S1: Richness of zebrafish-associated microbial communities.** Rarefaction curves are based on operational taxonomic units (OTUs) at a 97% sequence similarity threshold. Each data point refers to DNA extracted from 10 pooled zebrafishes (all samples from day 5, day 7, day14 and ZF21B2 and ZF21E2), or reverse transcribed RNA from 10 pooled zebrafish guts (ZF21B1R, ZF21B2R, ZF21E1R and ZF21E2R). Sample names are built up as follows: ZF=zebrafish; dpf (5, 7, 14 or 21); diet (p, pB, pE, B, E); replicate (1, 2); (R) if a sample is derived from RNA.


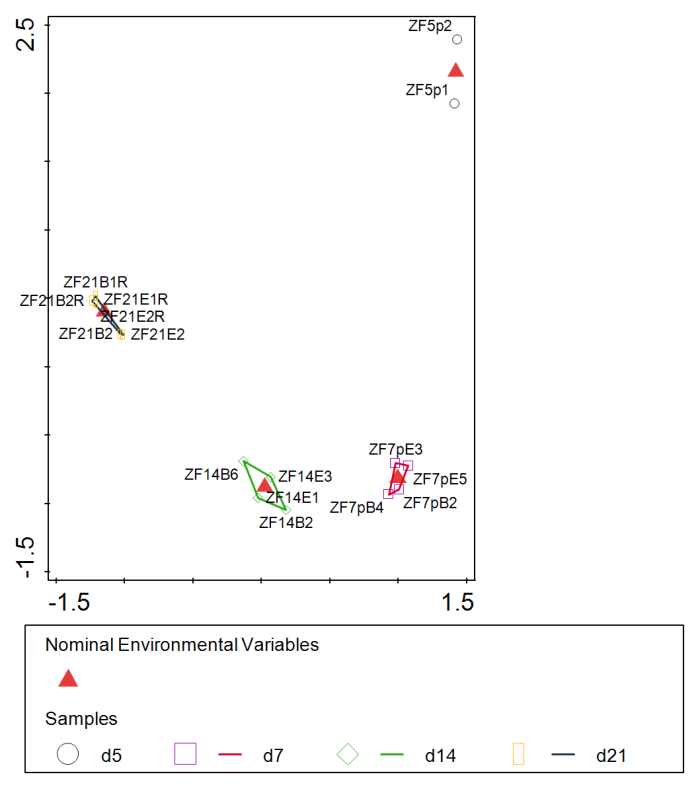


**7**

**5**

**21**

**14**

**CCA2**

**CCA1**

**Fig. S2: CCA-ordination plot of the zebrafish microbiota.** The red triangles represent the centroids of the datasets belonging to different time points indicated with the number in red. Each data point refers to DNA extracted from 10 pooled zebrafishes (all samples from day 5, day 7, day14 and ZF21B2 and ZF21E2), or reverse transcribed RNA from 10 pooled zebrafish guts (ZF21B1R, ZF21B2R, ZF21E1R and ZF21E2R). Sample names are built up as follows: ZF=zebrafish; dpf (5, 7, 14 or 21); diet (p, pB, pE, B, E); replicate (1, 2); (R) if a sample is derived from RNA.
